# 8-Way Randomized Controlled Trial of Doxylamine, Pyridoxine and Dicyclomine for Nausea and Vomiting during Pregnancy: Restoration of Unpublished Information

**DOI:** 10.1371/journal.pone.0167609

**Published:** 2017-01-04

**Authors:** Rujun Zhang, Navindra Persaud

**Affiliations:** 1 Department of Family and Community Medicine, University of Toronto, Toronto, Ontario, Canada; 2 Keenan Research Centre of the Li Ka Shing Knowledge Institute, St. Michael's Hospital, Toronto, Ontario, Canada; 3 Department of Family and Community Medicine, University of Toronto, Toronto, Ontario, Canada; the University of Sydney, AUSTRALIA

## Abstract

**Objectives:**

We report information about an unpublished 1970s study (“8-way” Bendectin Study) that aimed to evaluate the relative therapeutic efficacy of doxylamine, pyridoxine, and dicyclomine in the management of nausea and vomiting during pregnancy. We are publishing the trial's findings according to the restoring invisible and abandoned trials (RIAT) initiative because the trial was never published.

**Design:**

Double blinded, multi-centred, randomized placebo-controlled study.

**Setting:**

14 clinics in the United States.

**Participants:**

2308 patients in the first 12 weeks of pregnancy with complaints of nausea or vomiting were enrolled.

**Interventions:**

Each patient was randomized to one of eight arms: placebo, doxylamine/pyridoxine/dicylcomine, doxylamine/pyridoxine, dicylomine/pyridoxine, doxylamine, dicyclomine/pyridoxine, pyridoxine and dicyclomine. Each patient was instructed to take 2 tablets at bedtime and 1 additional tablet in the afternoon or morning if needed, for 7 nights.

**Outcomes:**

Reported outcomes included the number of hours of nausea reported by patients, the frequency of vomiting reported by patients and the overall efficacy of medication as judged by physicians.

**Results:**

Data from 1599 (69% of those randomized) participants were analyzed. Based on the available summary data of physician evaluation of symptoms and ignoring missing data and data integrity issues, the proportion of participants who were “evaluated moderate or excellent” was greater in each of the seven active treatment groups when compared with placebo (57%): doxylamine/pyridoxine/dicylcomine (14% absolute difference versus placebo; 95% CI: 4 to 24), doxylamine/pyridoxine (21; 95% CI 11 to 30), dicylomine/pyridoxine (21; 95% CI 11 to 30), doxylamine (20; 95% CI 10 to 29), dicyclomine/pyridoxine (4; 95% CI -6 to 14), pyridoxine (9; 95% CI -1 to 19) and dicyclomine (4; 95% CI -6 to 14). Based on incomplete information, the most common adverse events were apparently drowsiness and fatigue. There is a high risk of bias in these previously unpublished results given the high attrition rate in a 7 day trial, the lack of prespecified outcomes or analyses, and the exclusion of some data because of questionable data integrity.

**Conclusion:**

The available information about this “8-way Bendectin” trial indicates it should not be used to support the efficacy of doxylamine, pyridoxine or dicyclomine for the treatment of nausea and vomiting during pregnancy because of a high risk of bias.

**Trial registration:**

Not registered.

## Introduction

Nausea and vomiting are common symptoms experienced during pregnancy and may affect more than 80% of pregnant women [1, 2]. Nausea and vomiting during pregnancy (NVP) is self-limited and often resolves abruptly without treatment around the start of the second trimester [2]. Symptomatic treatments for NVP include dietary changes such as eating small meals frequently, non-pharmacological treatments such as ginger and pharmacological treatments including antiemetics that are used outside of pregnancy such as antihistamines and metoclopramide [3].

Doxylamine-pyridoxine is the first-line pharmacological therapy for NVP according to the current guideline by the Society of Obstetricians and Gynaecologist of Canada [4], Motherisk [5], and the American College of Obstetricians and Gynecologists [6]. One prescription for doxylamine-pyridoxine is filled for every two live births in Canada [7]. In contrast, antihistamines as a class are recommended for NVP in the United Kingdom [8].

There is limited published evidence for the efficacy of doxylamine and pyridoxine according to a recent Cochrane systematic review [3]. One unpublished study has been referred to in support of the use of the combination of doxylamine and pyridoxine. The “Bendectin Antinauseant 8-way” study was conducted during the 1970s and the Cochrane review was criticized for not including this study: “The Cochrane review did not provide complete information on the fact that this combination was approved by the FDA for use in the United States after a multi-arm RCT in the 1970s.” [9]. A narrative review of doxylamine-pyridoxine’s efficacy and safety for NVP in 2000 cites the unpublished 8-way study’s result, which “confirmed that the efficacy of Bendectin was greater than that of placebo but showed no contributions from dicyclomine in the association. Doxylamine was the major component, but pyridoxine had a clear effect on nausea but probably not vomiting” [10]. A 2014 article on the effectiveness and fetal safety of delayed-release combination of doxylamine and pyridoxine for the treatment of NVP references the unpublished study’s result: “Doxylamine most effective, dicyclomine no effect, pyridoxine effective for nausea but not vomiting” [11]. The website Bendectin.com (a website funded by Diclectin manufacturer Duchesnay Inc.) referenced the unpublished study as a factor in the reformulation of Bendectin: “Following an 8-way study, Bendectin was reformulated in 1976 to contain only the two active ingredients (doxylamine, pyridoxine) that demonstrated efficacy to treat NVP.” [12]. The United States Food and Drug Administration’s (US FDA’s) initial approval of Bendectin was apparently based on this trial: “The 1975 FDA Review of the ‘8-way’ study confirms that doxylamine alone and the combination of doxylamine and pyridoxine were effective in the control of nausea and vomiting of pregnancy” [12]. This 8-way trial was also considered in the US FDA’s recent approval of a new product containing doxylamine and pyridoxine because approval of combination products require that efficacy is demonstrated for each active ingredient [12]. The dose of doxylamine and pyridoxine used in the 8-way trial (10 mg each) is the same as that in the currently approved and recommended treatments.

Given the reliance on this unpublished trial and the current common usage of doxylamine and pyridoxine, we are publishing information about this trial according to the restoring invisible and abandoned trials (RIAT) initiative [13].

## Methods

### Restoring invisible and abandoned trials (RIAT) protocol

This paper adheres to the reporting standards that ensure accountability outlined in the RIAT initiative methods [13]. In order to obtain information about this trial, we made freedom of information requests to the United States Food and Drug Administration (US FDA) (13 April 2015), Health Canada (6 September 2011) and the European Medicines Agencies (29 April 2015). We also contacted the Bendectin Peer Group as well as several other individuals with experience obtaining unpublished data. We reviewed approximately 36 000 pages of information received from the FDA and approximately 7 200 pages were related to this trial. (The other pages were apparently about other studies, malformation reports and various correspondences. Some pages contained information that was difficult to read as apparently the information was stored on microfiches). After reviewing the information from the US FDA, we called the FDA to ask if any higher quality reproductions were available or if any further information (such as subsequent submissions) was available and we were told that we were sent all information relevant to our broad request. We also obtained 359 pages of information from Health Canada but 212 pages were redacted. The European Medicines Agency informed us that they had no relevant records related to our request. We received no relevant information from Duchesnay following our request.

We were unable to contact any of the site investigators listed in the report after searching for their contact information online; we found some indications that many of the investigators have since died. The Bendectin Peer Group, who were assembled to review the study for the US FDA, were notified by the restorative authors via fax on August 3, 2013 and were asked to respond in 30 days. None declared any intention to restore the record (appendix A). On 26 September 2013, as per the RIAT initiative methods [13], we posted to the BMJ website a rapid response publicly declaring our intent to publish the trial's findings: "Following the publication of the Restoring invisible and abandoned trials (RIAT) paper, we notified 4 members of the Bendectin Peer Group on 8 August 2013 that we intend to publish the results of the unpublished 1970s study on the efficacy of doxylamine and pyridoxine (“8-way” Bendectin Study). As of 25 September 2013, 3 did not respond to our letter and 1 responded but did not register intent to publish the trial. We are now publicly declaring our intention to publish the findings of this clinical trial." No response was received.

The original report of the Bendectin Peer Group study is included in appendix B. In addition, the RIAT audit record (RIATAR), which documents the sources of all information is also included in appendix C.

The description of the design and conduct of the study below is based on the information we reviewed.

### Design

The study used a double blind, multi-centred, randomized controlled study design. 14 clinics in the United States were involved. Factorial analyses were performed. No sample size calculation was reported.

### Participants

Eligibility criteria included the following: women in the first trimester of pregnancy (first 12 weeks of gestation), complaining of nausea and/or vomiting, and “only those who, in the opinion of the investigator, will be cooperative and complete the questionnaires”.

### Interventions

Each patient in the study was randomized into 1 of the following 8 interventions: (1) 10 mg dicyclomine hydrochloride (Bentyl), (2) 10 mg doxylamine succinate (Decapryn), (3) dicyclominehydrocholride/doxylamine succinate combination (10 mg each), (4) placebo, (5) 10 mg pyridoxine hydrochloride. (6) dicyclomine hydrochloride/ pyridoxine succinate combination (10 mg each), (7) Doxylamine succinate/ pyridoxine succinate combination (10 mg each), (8) dicyclomine hydrochloride/ doxylamine succinate/ pyridoxine hydrochloride combination (10 mg each) (Bendectin). Each patient was instructed to take 2 tablets at bedtime and, if necessary, 1 additional tablet in the morning and in the midafternoon, for 7 nights.

### Outcomes

Reported outcomes included physician judgements of efficacy (excellent, moderate, slight, or none), the number of hours of nausea reported on the patients’ daily diary cards, and the frequency of vomiting reported on the patients’ daily diary cards. The protocol does not indicate which outcome was the primary outcome and the trial was done before trials were registered prospectively.

The data were collected from the patient’s “In Dr.’s Office” or baseline daily diary card, 7-day self-report and the investigator’s initial and final evaluations. During the initial visit, the investigator completed and recorded the initial patient evaluation. Each patient was given the baseline card to complete immediately and 7 daily report forms to be completed each day of the intervention. The importance of completing the form promptly and not waiting until the end of the study to complete all forms was emphasized. At the end of the 7 days of intervention, the patients returned to the investigator who checked the forms for completeness, obtained any additional information and completed a final evaluation. The FDA- required Drug Experience Form (FD-1639) was to be completed for every patient who experienced serious side effects. Adverse events were recorded by physicians when volunteered by participants.

“Study completed according to protocol” is defined as availability of 7 or 8 patient diary cards (including the baseline card) with one or more check marks in each part of each diary card.

Reasons for noncompliance were recorded. Adverse events were apparently recorded in the physician evaluation form.

### Randomization

During the initial visit, the investigator enrolled patients into the study and obtained consent. To ensure allocation concealment, a centralised service at Merrell-National Laboratories was used. Medications were identical in appearance and had the coating used for Bendectin. Each medication was packaged in bottles of 30 tablets and each bottle was labeled with a tear-off label. The sealed tear-off portion contained the identity of the contents. The randomized intervention was to be revealed only after serious problem arose, and after contacting the project monitor at Merrell-National Laboratories.

### Statistical analysis

We report here the findings and one-sided p-values as they were originally reported.

The data were analysed by the Biostatisical Department of Merrell-National Laboratories. The statistical methods were not reported. Wherever possible, we also present our estimate of the same results. As original results were presented as percentages, without denominators or numerical results, we used information available elsewhere in the trial report to estimate denominators for each treatment arm and to calculate exact numbers of women with specific outcomes based on reported percentages. Where we have relied on estimates rather than directly reported data, we have identified estimates as such in the relevant tables. We assume p-values less than 0.05 are statistically significant and we did not correct for multiple comparisons.

For our estimate of the difference between treatment groups based on available summary information, we used an “N -1” chi-squared implemented by MedCalc (Belgium). [14, 15] Complete individual participant level data were not available.

### Risk of bias

We used the Cochrane Risk of Bias tool to assess the quality of the trial [16]. The two authors each independently made assessments and discussed disagreements. Further changes were made during the peer review process.

### Registration

The trial was conducted before trial registration was routine. We were unable to retrospectively register this trial because the available information collected was insufficient. The Institutional Review Board details were not available.

## Results

### Information received

We obtained from the US FDA the original study report including the protocol and summary results, completed data collection forms for less than 25% of participants and correspondences regarding the trial and its review by the FDA (Appendix B).” We were unable to obtain the final study findings as the report used in this publication is apparently an interim one: “This submission contains information on case reports received through July 22 1974. Information on case reports subsequent to this date will be supplied in a future submission.” We are not aware of any subsequent submissions. We report here the findings from the study that is currently referenced in the literature.

We did not obtain any additional clearly relevant information from other sources; a large portion of the information received from Health Canada was redacted.

Of the 32 initial clinician investigators of the study, 3 never started the study, 26 terminated or completed their porting, and 3 were still actively collecting data at the time the report was generated. The data for 30 patients recruited by one of the investigators (who was one of the three recorded as actively collecting data) were excluded from the study following receipt of a March 19, 1975 letter from the Commissioner of Food and Drugs: “We find instances of overstatement of study duration; of data recording in absence of patient visits; instances where doubt exists concerning the identification of the product under investigation; and instances of non-reporting of the occurrence of 'pregnancies during the course of a contraceptive study.”

The results from the original study include the following information for each arm of treatment: severity of nausea or vomiting, prevalence of side effects, overall effectiveness of medication, the number of patients allocated, lost to follow up and completed per protocol.

### Trial results

**Fig 1:**
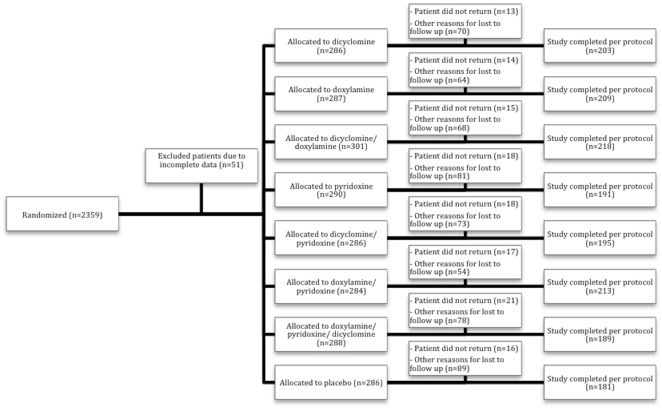
Participant flow diagram. Numbers of participants who were randomized, received intended treatment and were analyzed for the efficacy outcomes

The number assessed for eligibility is unknown. 2359 patients were initially enrolled and randomized in the study. 51 patients had unspecified “incomplete data” and were excluded from the study population after randomization. Of the remaining 2308 patients for whom some baseline characteristics are available (Table 1), 132 (6%) participants did not complete the study and were classified as “no return” or lost to follow up. 709 (30%) patients failed to meet protocol criteria and were excluded from the study (see S1 Table for reasons), leaving 1599 (65.8% of allocated) participants. Data about adverse events were missing for an additional 18 participants. The number randomized to each group was not reported but allocation seems to have been roughly even. (Fig 1) We could not find information about typical baseline characteristics (age, parity, duration of pregnancy at enrollment). Baseline symptoms are shown in Table 1.

**Table 1:** Baseline nausea and vomiting severity.

**Baseline nausea severity**					
	**None**	**Mild**	**Moderate**	**Severe**	**Not stated**
**doxylamine/pyridoxine/dicyclomine (n = 284)**	1 (0.4%)	67 (24%)	154 (54%)	61 (21%)	1 (0.4%)
**doxylamine/ pyridoxine (n = 279)**	0	50 (18%)	147 (53%)	81 (29%)	1 (0.4%)
**dicyclomine/ doxylamine (n = 295)**	0	55 (19%)	180 (61%)	59 (20%)	1 (0.3%)
**doxylamine (n = 283)**	0	66 (23%)	153 (54%)	64 (23%)	0
**dicyclomine/ pyridoxine (n = 281)**	0	66 (23%)	147 (52%)	68 (24%)	0
**pyridoxine (n = 286)**	1 (0.3%)	55 (19%)	150 (52%)	80 (28%)	0
**dicyclomine (n = 280)**	1 (0.4%)	60 (21%)	141 (50%)	77 (27%)	1 (0.4%)
**placebo (n = 281)**	0	64 (23%)	143 (51%)	74 (26%)	0
**Baseline vomiting severity**					
	**None**	**Mild**	**Moderate**	**Severe**	**Not stated**
**doxylamine/pyridoxine/dicyclomine (n = 284)**	133 (47%)	75 (26%)	56 (20%)	20 (7%)	0
**doxylamine/ pyridoxine (n = 279)**	122 (44%)	71 (25%)	59 (21%)	26 (9%)	1 (0.4%)
**dicyclomine/ doxylamine (n = 295)**	106 (36%)	80 (27%)	79 (27%)	30 (10%)	0
**doxylamine (n = 283)**	124 (44%)	83 (29%)	55 (19%)	20 (7%)	1 (0.4%)
**dicyclomine/ pyridoxine (n = 281)**	130 (46%)	81 (29%)	52 (19%)	18 (6%)	0
**pyridoxine (n = 286)**	124 (43%)	67 (23%)	66 (23%)	29 (10%)	0
**dicyclomine (n = 280)**	131 (47%)	64 (23%)	62 (22%)	22 (8%)	1 (0.4%)
**placebo (n = 281)**	104 (37%)	88 (31%)	64 (23%)	25 (9%)	0

Denominators were not provided in the review report.

We did not find information about adherence to trial interventions in the US FDA documents.

The difference between groups in symptom improvements according to physician evaluation (Table 2) and patient symptoms diaries (Table 3) were summarized in the review report. The results are reproduced from the review report and show one-sided p-values and no denominators. In Table 3, we could not determine how the “percent reduction from pre-treatment” was calculated.

**Table 2:** Symptom improvements as summarized in the study report based on physician’s evaluations.

Treatment	Effectiveness of Medication		Nausea		Vomiting	
	Percentage evaluated moderate or excellent (%)	p	Percentage improved (%)	p	Percentage improved (%)	p
**doxylamine/pyridoxine/dicyclomine**	71	<0.01	65	<0.01	77	0.03
**doxylamine/ pyridoxine**	78	<0.01	75	<0.01	73	0.17
**dicyclomine/ doxylamine**	78	<0.01	71	<0.01	74	0.07
**doxylamine**	77	<0.01	69	<0.01	78	0.01
**dicyclomine/ pyridoxine**	61	0.28	57	0.03	62	0.64
**pyridoxine**	66	0.10	68	<0.01	66	0.36
**dicyclomine**	61	0.17	61	0.07	71	0.33
**placebo**	57	-	52	-	66	-

The data are from all investigators (including the investigator whose data were subsequently excluded). The p values are one-sided probability based on tests of each active treatment versus placebo. The analysis of vomiting includes only those patients with vomiting symptoms at pre-treatment.

**Table 3:** Symptom improvements as summarized in the study report based on participant diary entries.

Treatment	Nausea		Vomiting	
	Percent reduction from pre-treatment	p	Percentage with no vomiting on 5 or more treatment days	p
**doxylamine/pyridoxine/dicyclomine**	57	<0.01	46	<0.01
**doxylamine/pyridoxine**	64	<0.01	48	<0.01
**dicyclomine/doxylamine**	50	<0.01	49	<0.01
**doxylamine**	56	<0.01	54	<0.01
**dicyclomine/pyridoxine**	44	0.03	39	0.08
**pyridoxine**	35	0.09	29	0.37
**dicyclomine**	36	0.25	30	0.26
**placebo**	31	-	28	-

The p values are one-sided probability based on tests of each active medication vs. placebo. The analysis of vomiting includes only those patients with vomiting symptoms at pre-treatment.

We estimated the differences between groups using the available summary information about physician evaluations (Tables 4 and 5). We assumed that the number of participants in each group was equal to those who completed the study per protocol and we assumed the reported percentage improvements shown in Tables 2 and 3. We disregarded the missing data for the purposes of the calculation.

**Table 4:** Estimated differences between treatment groups and placebo in physician evaluations of nausea based on available summary information for analyzed participants.

	Allocated n = 2308	Excluded, estimated missing (%) n = 709 (31%)	Analyzed n = 1599	reported percent improved	Absolute difference in % improved versus placebo (95% CI)
**doxylamine/pyridoxine/dicyclomine**	288	99 (34%)	189	71	14 (3.8 to 24)
**doxylamine/pyridoxine**	284	71 (25%)	213	78	21 (11 to 30)
**dicyclomine/doxylamine**	301	83 (28%)	218	78	21 (11 to 30)
**doxylamine**	287	78 (27%)	209	77	20 (10 to 29)
**dicyclomine/pyridoxine**	286	91 (32%)	195	61	4 (-6 to 14)
**pyridoxine**	290	99 (34%)	191	66	9 (-1.3 to 19)
**dicyclomine**	286	83 (29%)	203	61	4 (-6 to 14)
**placebo**	286	105 (37%)	181	57	-

**Table 5:** Nausea: reanalysis of patient diary reports of improvement in per protocol population.

	Per protocol population* (n), n = 1599	Estimated number (%) improved	Estimated relative risk of improvement versus placebo (95% CI)	Estimated absolute difference in % improvement versus placebo (95% CI)
**doxylamine/pyridoxine/ dicyclomine**	189	108 (57%)	1.8 (1.4 to 2.4)	26 (16 to 36)
**doxylamine/pyridoxine**	213	136 (64%)	2.1 (1.6 to 2.6)	33 (23 to 42)
**doxylamine/ dicyclomine**	218	109 (50%)	1.6 (1.2 to 2.1)	19 (9 to 29)
**doxylamine**	209	117 (56%)	1.8 (1.4 to 2.3)	25 (15 to 34)
**dicyclomine/pyridoxine**	195	86 (44%)	1.4 (1.1 to 1.9)	13 (2.8 to 23)
**pyridoxine**	191	67 (35%)	1.1 (0.85 to 1.5)	4 (-6.0 to 14)
**dicyclomine**	203	73 (36%)	1.2 (0.87 to 1.5)	5 (-4.8 to 15)
**placebo**	181	56 (31%)	-	-

*3 women had no nausea at baseline and were excluded from the nausea analysis

The results of some factorial analyses comparing the three combinations containing each constitutent the combination not containing that constituent were reported: combinations containing doxylamine were superior by both physician records and patient reports (p < 0.01); combinations containing pyridoxine were superior according to patient reports (p < 0.01) but the difference was not statistically significant based on physician records (p = 0.08); and for dicyclomine factorial analyses were not reported although there was a comment that “the contribution of dicyclomine to the efficacy of doxylamine when given in combination was not measurable in this study”.

### Adverse effects

Adverse event information was reportedly available for 2158 participants (93%). No serious adverse effects were reported for any of the medications used in the study. The total number of adverse events and the incidence of the most common adverse events reported are shown in Table 6.

**Table 6:** Total adverse events and common (>1% above placebo) adverse events reported.

	Adverse event	Drowsiness	Fatigue	Headache
**doxylamine/pyridoxine/dicyclomine (n = 267)**	38 (14%)	12 (4.5%)	5 (1.9%)	4 (1.5%)
**doxylamine/ pyridoxine (n = 267)**	23 (9%)	15 (5.6%)	2 (0.7%)	2 (0.7%)
**dicyclomine/ doxylamine (n = 286)**	39 (14%)	15 (5.2%)	7 (2.4%)	8 (2.8%)
**doxylamine (n = 273)**	41 (15%)	14 (5.1%)	6 (2.2%)	6 (2.2%)
**dicyclomine/ pyridoxine (n = 266)**	32 (12%)	3 (1.1%)	5 (1.9%)	10 (3.8%)
**pyridoxine (n = 272)**	26 (10%)	3 (1.1%)	1 (0.4%)	5 (1.8%)
**dicyclomine (n = 273)**	29 (11%)	4 (1.5%)	4 (1.5%)	9 (3.3%)
**placebo (n = 270)**	30 (11%)	8 (3.0%)	3 (1.1%)	4 (1.5%)

Percentages are based on the number allocated per treatment arm

### Risk of bias

The risk of bias was high in several domains (Table 7). The trial predated reporting guidelines and information about several domains was incomplete.

**Table 7:** Cochrane Collaboration Risk of Bias tool.

Domain	Authors judgment	Support for judgment
**Sequence Generation (selection bias)**	Unclear	The investigators do not describe the sequence generation process. It is unclear from the available information whether sequence generation was random.
**Allocation Concealment (selection bias)**	Unclear	Participants and investigators enrolling participants could not foresee assignment because a centralized service at Merrell-National Laboratories was used to allocate the study groups. Typical baseline characteristics were not reported.
**Blinding of participants and researchers (performance bias)**	Low	The medications and bottles were identical in appearance. Each medication was packaged in bottles of 30 tablets and each bottle was labeled with a tear-off label. The sealed-off portion contained the identity of the contents.
**Blinding of outcome assessment (detection bias)**	Low	The outcome assessors (physicians from multiple centers and participants) were blinded to the intervention. It is unclear if data analysts were blinded.
**Incomplete outcome data (attrition bias)**	High	Apparently results were analyzed for 1599 (68%) of 2359. 51 of the 2359 participants were initially excluded from the study due to “incomplete data”. Of the remaining participants, 6% did not complete the study and were classified as lost to follow up and 30% failed to meet protocol criteria and were excluded from the study. In addition, information about adverse outcomes are missing for a small fraction of analysed participants.
**Selective outcome reporting (reporting bias)**	High	No outcomes were prespecified. The trial was done before outcomes were registered. Multiple outcomes are reported without identifying any as primary.
**Other potential threats to validity**	High	Important information about the study is not available. The FDA ordered that data from one investigator be excluded because of concerns about data integrity. The trial was apparently not completed. The results were never published.

## Discussion

This previously unpublished 1970s trial of medications for NVP that has been cited as supporting the use of doxylamine-pyridoxine has several important limitations: the final results of the study are not available, data from more than 30% of recruited individuals were not analyzed although the follow up period was only one week, few details about outcome determination or statistical analyses were provided. The integrity of the data is questionable because data from at least one investigator were subsequently excluded for reasons including “data recording in absence of patient visits”. The data known to be of questionable integrity was a small fraction of the data included in the available interim analysis and we do not know if problems with the integrity of other data were identified, the reason the final results of the trial are not available or the reason these results were not published previously. The risk of bias was high in several domains. Also, limited baseline data is available, the number of tablets taken by each participant is not available and that number could have doubled based on as needed use, the method by which physicians scored symptoms was not clear, outcome data is unavailable for 37% of participants in the placebo arm that was used as the reference for comparisons, p-values were one-sided and not corrected for the multiple comparisons made, the approach to accounting for the effect of missing data is not described and some information about adverse events may be missing.

The US FDA review report for the 8-way study concluded that the combination of doxylamine, pyridoxine and dicyclomine is efficacious in the treatment of NVP and that the efficacious components are likely doxylamine and pyridoxine but not dicyclomine. Several sources have cited this trial in support of doxylamine and pyridoxine for the treatment of nausea and vomiting during pregnancy [9–12, 17]. In 2013, the US FDA referred to the results of this trial in approving a product containing doxylamine and pyridoxine: “…this reviewer concurs that the 1975 ‘8-way’ study supports the effectiveness of Diclegis in the treatment of NVP.” This recent US FDA review quoted the results of this 8-way study (e.g. “The control of nausea by doxylamine alone and by each of the 3 combination which contain doxylamine was consistently statistically significantly (p<0.01) superior to placebo by both physician’s records and patient’s records”) but did not mention any of the problems with the study described here, not even the problem with data integrity that was originally identified by the US FDA. Some parts of the US FDA’s recent review are redacted. Health Canada currently relies on this trial in support of the efficacy of doxylamine and pyridoxine [18]. A 2015 Cochrane review [3] concluded there was insufficient evidence supporting the efficacy of any particular intervention including doxylamine-pyridoxine. The information presented here including the risk of bias assessment may be used to decide whether this trial should be included in future systematic reviews.

This manuscript and the supporting materials make information about this previously unpublished trial easily available for consideration by regulators, researchers, clinicians and patients. Despite our efforts over several years to obtain available information from regulators and others who might have information about the study, the utility of publishing this information is limited by the lack of some important information including individual participant level data. Instead of using individual participant level data, we estimated differences between groups using summary data and based on several assumptions. Other details of the protocol such as how clinicians judged clinical response or the statistical tests performed could not be obtained.

## Conclusion

The information published here for the first time about this 1970s trial allow for an assessment of the evidence base supporting the use of commonly used agents for nausea and vomiting of pregnancy: doxylamine and pyridoxine. While the analyzed data indicate differences from placebo for several combinations, the questionable data integrity, high drop-out rate, and other methodological concerns mean that the prescribing of this medication should not be based on this trial. No firm conclusion about the efficacy or safety of doxylamine or pyridoxine can be drawn from the limited available information. The claims about the efficacy of doxylamine and pyridoxine and the clinical practice guidelines and regulatory decisions that are based on this trial should be revisited. All of the available information about clinical trials of treatments for nausea and vomiting during pregnancy should be made publicly available so that informed decisions can be made by regulators, researchers, clinicians and patients.

## Supporting Information

S1 Table Primary reason for dropout for 425 participants (60% of 709 dropouts) provided in US FDA documents. The source of the information is the US FDA fiche 353, page 4. For the remaining 284 dropouts, the reasons included: (1) one or more diary cards missing, (2) no study medication taken 1 or more days, (3) interval of 1 day or more between visit to physician and starting medication or between diary dates, (4) anti-emetic other than study medication taken at any time during the study, or (5) miscellaneous. Of these 284, 33 “could not definitely be classes as dropouts; their status was questionable” (and these participants were “included with the 251 patients who did not fulfill protocol criteria for other reasons”).
(DOCX)

S1 Appendix Correspondences with US FDA reviewers. (DOCX)

S2 Appendix US FDA review of submissions. (PDF)

S3 Appendix RIAT Audit Record. A tool based on the CONSORT 2010 checklist, for documenting the transformation from regulatory documents to journal publication.
(DOCX)
