# Supplementary material for: 8-Way Randomized Controlled Trial of Doxylamine, Pyridoxine and Dicyclomine for Nausea and Vomiting during Pregnancy: Restoration of Unpublished Information
Source: PLoS One. 2017 Jan 4;12(1):e0167609. doi: 10.1371/journal.pone.0167609 (PMC5215753; doi:10.1371/journal.pone.0167609)
Supplement: S1 Table — The source of the information is the US FDA fiche 353, page 4. For the remaining 284 dropouts, the reasons included: (1) one or more diary cards missing, (2) no study medication taken 1 or more days, (3) interval of 1 day or more between visit to physician and starting medication or between diary dates, (4) anti-emetic other than study medication taken at any time during the study, or (5) miscellaneous. Of these 284, 33 “could not definitely be classes as dropouts; their status was questionable” (and these participants were “included with the 251 patients who did not fulfill protocol criteria for other reasons”). (DOCX) [file pone.0167609.s001.docx]

***Supplementary Table 1.*** Primary reason for dropout for 425 participants (60 % of 709 dropouts) provided in US FDA documents

|  | Lack of efficacy | Adverse events | Loss of symptoms | *Label not provided in US FDA Document* | *Label not provided in US FDA Document* | *Label not provided in US FDA Document* | Did not attend final evaluation | Total |
| --- | --- | --- | --- | --- | --- | --- | --- | --- |
| doxylamine/pyridoxine/dicyclomine | 9 | 5 | 7 | 2 | 0 | 13 | 21 | 57 |
| doxylamine/pyridoxine | 5 | 7 | 3 | 0 | 1 | 11 | 17 | 44 |
| dicyclomine/doxylamine | 3 | 3 | 6 | 0 | 3 | 12 | 15 | 42 |
| doxylamine | 3 | 8 | 0 | 3 | 0 | 11 | 14 | 39 |
| dicyclomine/pyridoxine | 27 | 5 | 3 | 3 | 2 | 9 | 18 | 67 |
| pyridoxine | 16 | 2 | 5 | 0 | 1 | 12 | 18 | 54 |
| dicyclomine | 20 | 3 | 2 | 2 | 0 | 13 | 13 | 53 |
| placebo | 29 | 7 | 2 | 0 | 2 | 13 | 16 | 69 |
| Total | 112 | 40 | 28 | 10 | 9 | 94 | 132 | 425 |

The source of the information is the US FDA fiche 353, page 4. For the remaining 284 dropouts, the reasons included: (1) one or more diary cards missing, (2) no study medication taken 1 or more days, (3) interval of 1 day or more between visit to physician and starting medication or between diary dates, (4) anti-emetic other than study medication taken at any time during the study, or (5) miscellaneous. Of these 284, 33 “could not definitely be classes as dropouts; their status was questionable” (and these participants were “included with the 251 patients who did not fulfill protocol criteria for other reasons”).
