# Supplementary material for: 8-Way Randomized Controlled Trial of Doxylamine, Pyridoxine and Dicyclomine for Nausea and Vomiting during Pregnancy: Restoration of Unpublished Information
Source: PLoS One. 2017 Jan 4;12(1):e0167609. doi: 10.1371/journal.pone.0167609 (PMC5215753; doi:10.1371/journal.pone.0167609)
Supplement: S1 Appendix — (DOCX) [file pone.0167609.s002.docx]

**S1 APPENDIX**

**Letter 1- Fax to Robert Brent (Original author).**

**
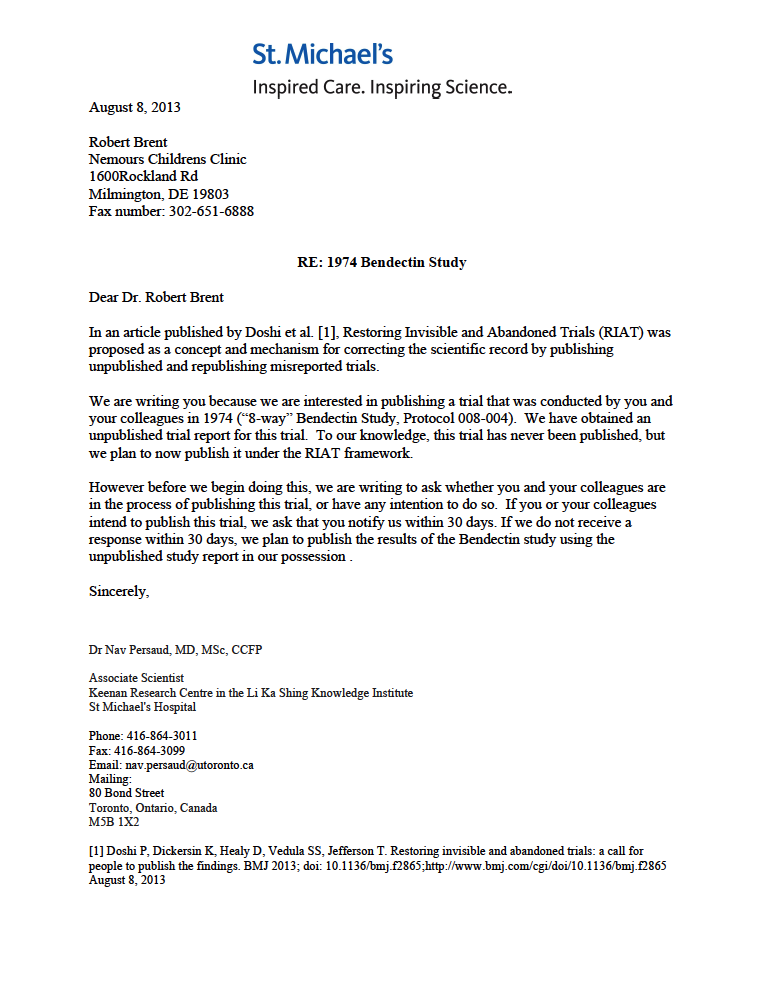
**

**Letter 2- Fax to John Hobbins (Original author)**


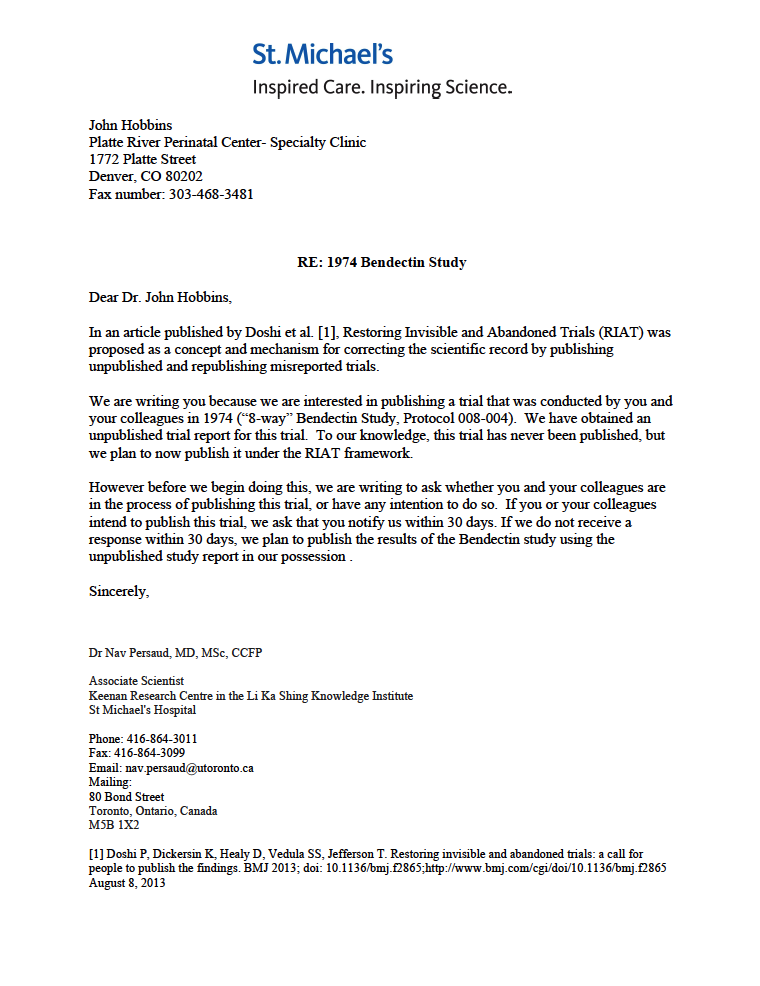


**Letter 3- Response by Robert Brent (Original author).**

**
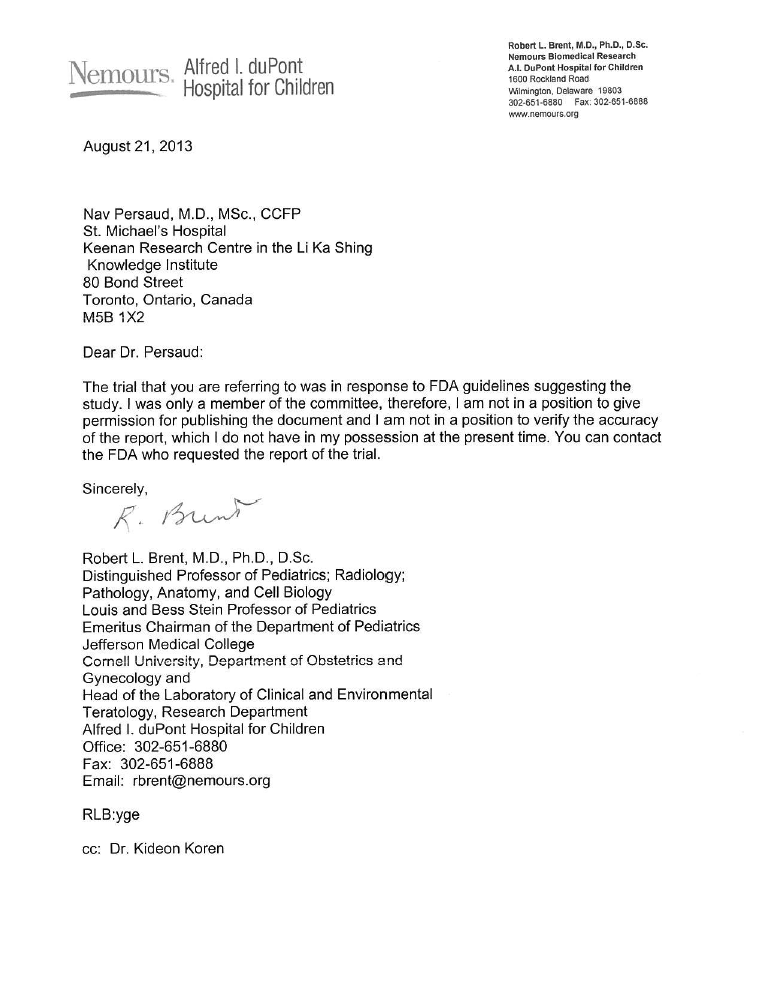
**
