# Supplementary material for: 8-Way Randomized Controlled Trial of Doxylamine, Pyridoxine and Dicyclomine for Nausea and Vomiting during Pregnancy: Restoration of Unpublished Information
Source: PLoS One. 2017 Jan 4;12(1):e0167609. doi: 10.1371/journal.pone.0167609 (PMC5215753; doi:10.1371/journal.pone.0167609)
Supplement: S2 Appendix — (PDF) [file pone.0167609.s003.pdf]

Bendectin

NDA-10-598

(DESI 10598)

Dicyclomine/Doxylamine (Bentyl/Decapryn)

(IND [illegible]620)

OVERALL SUMMARY OF "3-WAY" BENDECTIN STUDY (PROTOCOL 008- _ -004)

REVIEW OF SUBMISSIONS (1972 THROUGH 1974)--HISTORICAL

Bendectin is a combination product for nausea and vomiting of pregnancy. It consists of an anticholinergic spasmolytic (dicyclomine hydrochloride--Bentyl), an antihistamine (doxylamine succinate--Decapryn), and vitamin B_6 (pyridoxine hydrochloride). Each tablet of Bendectin contains 10 mg. of each of the 3 ingredients.

An overall summary and IBM print-outs related primarily to support of the efficacy of Bendectin and 2 of its ingredients (dicyclomine and doxylamine) for nausea and vomiting of pregnancy were submitted September 26, 1972. The 16 volumes of that submission bear a September 25, 1972 date, and were summarized in Volume 2 thereof. Volumes 2 and 5 through 13 referred to the then-recently-completed "4-way" multi-center study comparing dicyclomine, doxylamine, dicyclomine/doxylamine, and placebo, and Volumes 1 through 4 referred to the previously completed and reported (including submission of case reports) multi-center Bendectin vs. placebo studies (submissions of 7/30/68; 7/30/69; 10/22/70; and 3/6/72). Volumes 14 and 15 referred to literature reviews on the importance of pyridoxine as related to: a) its influence on metabolic indices during pregnancy, and

b) its effect, or the effect of its deficiency, on the developing fetus or resulting progeny, respectively. Volume 16 summarized the cases of reported congenitally malformed infants born of patients who had received Bendectin during pregnancy.

The earlier 10/22/70 and 3/6/72 submissions provided statistical evidence for the significant efficacy of Bendectin therapy over placebo therapy. The 9/26/72 submission, supplemented by the later submissions of 12/15/72, 6/18/73, and 10/8/73 (statistical only), provided evidence for the efficacy of doxylamine alone and in combination with dicyclomine; the efficacy for dicyclomine was suggestive but uncertain.

The individual case reports for the dicyclomine/doxylamine ("4-way") study upon which the 9/26/72 submission was based were included in Volumes 2 through 28 of the 37 volumes bearing the date of 12/5/72, and submitted 12/15/72. Volume 1 provided an introduction, general summary, and other general information related to protocol, investigators, and patients. The additional and untabulated (excluded from analysis) case reports from the dicyclomine/doxylamine ("4-way") study were in Volumes 29 and 30. Adverse reactions reported in the dicyclomine/doxylamine (volunteered and solicited) and the earlier Bendectin (volunteered only) studies were summarized in Volume 31, with the FD-1639's from the dicyclomine/doxylamine study in Volumes 32 through 36 of that submission. Volume 37 provided case report data for the congenital malformations summarized in Volume 16 of the 9/26/72 submission.

03/14/75

Comments from consultants supporting the safety of Bendectin as related to birth defects of infants born of mothers who took Bendectin during pregnancy were submitted on 5/30/73. Comments from consultants supporting the efficacy of Bendectin and of the 2 ingredients (dicyclomine and doxylamine) were submitted on 6/15/73 and 7/2/73, respectively.

SUMMARY OF "8-WAY" MULTI-CENTER STUDY*--THIS SUBMISSION

Introduction.

This current submission is a report of the results of a randomized, parallel, double-blind, multi-center study of Bendectin involving 29 investigators and a total population of 2308 patients on whom a physician's initial and final evaluation record were obtained, and who were pregnant, had record of having taken identifiable medication, and had nausea and/or vomiting of pregnancy as indicated on the physician's initial evaluation form. Bendectin, each of its ingredients alone and in combination were compared with placebo in an "8-way" study design. The 8 medications were: dicyclomine, doxylamine, dicyclomine/doxylamine, pyridoxine, dicyclomine/pyridoxine, doxylamine/pyridoxine, Bendectin, and placebo. The front of the volumes contains the table of contents for the entire submission.

*Portions of this summary duplicate that presented in Volume 1, but only in Volume 1 are the results of the current "8-way" study brought into perspective with information previously known about this drug.

Volume 1 of this submission contains an overall summary of Bendectin, including an overall sponsor's summary of the "8-way" study now completed, and other general or summary information. Volume 2 (this volume) contains further details concerning protocol, investigators, patient population, and adverse reactions reported (volunteered) during this "8-way" investigation. Volume 3 is a report of the biostatistical analysis of this study. The subsequent volumes provide copies of IBM print-outs and of original case reports, as identified in "contents" in the front of the volumes. Summaries of the status of the studies of the individual investigators are included in the volumes bearing the case reports and precede each investigator's first case report.

Summary of Protocol.

Participants in this study were volunteer obstetric patients with nausea and/or vomiting of pregnancy (complete protocol in this volume).

Each patient was given 30 tablets of medication and instructed to take 2 tablets at bedtime for 7 nights, and if necessary 1 additional tablet in the mornings and/or midafternoons. Evaluations by the investigator were performed at the time of initial visit and again following completion of treatment. The patients completed a diary card form before therapy and on each of the study days, and returned these to the investigator after the medication period. Therapeutic efficacy was evaluated by a series of parameters, including hours of nausea and frequency of vomiting as

reported on the patients' daily diary cards, and an overall effectiveness of medication judgment by the physician. The data were tabulated and subjected to statistical analyses. Adverse reactions volunteered by the patients were recorded.

Summary of Results.

Data were obtained, tabulated, and summarized on a total of 2308 patients on whom the physician's initial and final evaluation record were obtained and who had record of having taken identifiable medication, were pregnant, and had nausea and/or vomiting of pregnancy as indicated on the physician's initial evaluation form. (Incomplete data were obtained on an additional untabulated 51 patients who were excluded from the 2308 population.)* A total of 1599 patients with nausea and/or vomiting reported that they took medication on each of the 6 successive study days and supplied diary cards for pretreatment and each of these 6 days. (The reasons for 709 patients failing to meet protocol criteria are summarized by a statement later in this volume.) Table 1 (Physician's Evaluation) and Table 2 (Patient's Diary Card) of the succeeding 2 pages summarize the primary statistical analysis of the results of the 8 medications on nausea and vomiting in this population of 1599 patients.+

*See end of later section in this volume presented as Summary of Explanations for Patients Not Meeting Protocol Criteria for paragraph entitled Untabulated Data.

+4/1/75: Reanalysis to exclude 30 patient reports from Dr. Balin results in no essential change in the figures in Tables 1 and 2. (See this volume, p. 11 ff.)

03/14/75

Table 1

Summary Table

Physician's Evaluation

Treatment	Effectiveness of Medication Percentage evaluated moderate or excellent	p	Nausea Percentage improved	p	Vomiting Percentage improved	p
Bendectin	71	<.01	65	<.01	77	.03
Doxylamine/ pyridoxine	78	<.01	75	<.01	73	.17
Dicyclomine/ doxylamine	78	<.01	71	<.01	74	.07
Doxylamine	77	<.01	69	<.01	78	.01
Dicyclomine/ pyridoxine	61	.28	57	.03	62	.64
Pyridoxine	66	.10	68	<.01	66	.36
Dicyclomine	61	.17	61	.07	71	.33
Placebo	57		52		66	

The p values are one-sided probabilities based on tests of each active medication vs.. placebo.

The analysis of vomiting includes only those patients with vomiting symptoms at pretreatment.

Table 2

Summary Table

Patient's Diary Card

Treatment	Nausea: Percent reduction from pretreatment	p	Vomiting: Percentage with no vomiting on 5 or more treatment days	p
Bendectin	57	<.01	46	<.01
Doxylamine/ pyridoxine	64	<.01	48	<.01
Dicyclomine/ doxylamine	50	<.01	49	<.01
Doxylamine	56	<.01	54	<.01
Dicyclomine/ pyridoxine	44	.03	39	.08
Pyridoxine	35	.09	29	.37
Dicyclomine	36	.25	30	.26
Placebo	31		28	

The p values are one-sided probabilities based on tests of each active medication vs. placebo.

The analysis of vomiting includes only those patients with vomiting symptoms at pretreatment.

The biostatistical report (Volume 3) provides the complete and detailed statistical analysis of this 1599 patient population as well as the total 2308 patient population. Various parameters were observed and the variables were studied by biostatistical methods described therein. The following comments are based on the more pertinent data generated by this study.

The control of nausea by doxylamine alone and by each of the 3 combinations which contain doxylamine was consistently statistically significantly ($p < 0.01$) superior to placebo by both physicians' records and patients' records. Additionally, the control of vomiting favored all formulations containing doxylamine by a statistical significance, as compared to placebo, of $p < 0.01$ by the patients' records and in 2 of the 4 doxylamine formulations (i.e., doxylamine alone and Bendectin) of $p \leq 0.03$ by the physicians' records. By factorial analysis, all medications with doxylamine alone or in combination (4 medications) were, by physicians' records and patients' records, more effective in controlling nausea and vomiting than those which did not contain this ingredient (4 medications) with a statistical probability of < 0.01.

Pyridoxine alone excelled over placebo ($p < 0.01$) in the reduction of nausea as demonstrated by physicians' records; the patients' records of nausea favored pyridoxine with $p = 0.09$. Greater efficacy for treatment of nausea by doxylamine/pyridoxine over doxylamine alone was supported marginally with p values of 0.12 and 0.26 by the patients' records and physicians' records, respectively. Factorial analysis of the 4 medications with vs. without pyridoxine indicated effectiveness in the control of nausea with p values of 0.01 by patients' records and 0.08 by physicians' records.

Dicyclomine alone had marginal efficacy over placebo by both physicians' records and patients' records in the treatment of nausea ($p = 0.07$ by physicians' records; $p = 0.25$ by patients' records). Dicyclomine combined with pyridoxine was superior to placebo ($p = 0.03$) for control of nausea by both patients' records and physicians' evaluations. The contribution of dicyclomine to the efficacy of doxylamine when given in combination was not measurable in this study.

No serious adverse effects were reported for any of the medications (full summary in this volume). The incidence of reported adverse effects among the medication groups varied form 8.7% for doxylamine/pyridoxine to 15.2% for doxylamine, with a mean of 12% for the total population. Drowsiness was an adverse effect relatable to one of the ingredients (doxylamine) of Bendectin. Patient groups taking medication which contained doxylamine had an incidence of drowsiness from 1.56% to 2.69% above placebo, whereas patients taking dicyclomine, pyridoxine, or a combination of dicyclomine/pyridoxine had an incidence of drowsiness of 1.42% or more below placebo. The incidence of drowsiness among placebo patients was 2.97%. Headache had a 1.34% to 2.37% greater incidence compared to placebo among patients taking dicyclomine, dicyclomine/doxylamine, or dicyclomine/pyridoxine, whereas all other medication groups showed an incidence of headache of < 1% difference from the placebo incidence of 1.49%.

Conclusions.

The following are conclusions of this "8-way" study deemed to be relevant to clinically significant actions:

1. This "8-way" study confirms the previous findings that Bendectin is effective in the control of nausea and vomiting of pregnancy.

2. This "8-way" study confirms the previous findings that doxylamine and the combinations containing doxylamine (including Bendectin) are effective in the control of nausea and vomiting of pregnancy.

3. Pyridoxine alone and pyridoxine combined with dicyclomine, which were not evaluated in the earlier "4-way" study, were by physicians' records more effective than placebo in the control of nausea in this study by a statistically significant degree. Factorial analysis of all groups with pyridoxine vs. treatment without by patients' records demonstrated a statistically significant contribution of pyridoxine to the control of nausea.

4. The rationale for providing pyridoxine as a nutritional supplement during pregnancy and in the dosage employed, plus the evidence of its efficacy for control of nausea as well as its contribution to the efficacy of the combination as demonstrated in this study, indicates that pyridoxine is a clinically important component of the anti-nausea/anti-emetic product, Bendectin.

5. Dicyclomine, in this study as in the earlier "4-way" study tended to be superior to placebo for control of nausea and/or vomiting, but not to a statistically significant degree.

03/14/75

6. The efficacy of Bendectin for nausea and vomiting of pregnancy was confirmed, and the contributions made by the 3 ingredients in decreasing order of demonstrability by this study were doxylamine - pyridoxine - dicyclomine. The difficulty in demonstrating a statistically significant contribution of pyridoxine and dicyclomine over the considerable efficacy of doxylamine does not negate the reality of their contribution.

Current Status.

Of the 32 clinicians who originally agreed to be investigators, 3 never started the study, 26 have terminated or completed their studies, and 3 are still active with their studies in progress (see listing of investigators in this volume). This submission contains information on the case reports received through July 22, 1974. Information received on case reports subsequent to this date will be supplied in a future submission. No adverse reactions were reported since 7/22/74 which were different in type or in incidence from those received prior to that date.

RE-EVALUATION OMITTING DATA FROM DR. BALIN (INV. NO. 113)--4/1/75

Following receipt of the March 19, 1975 letter from Alexander M. Schmidt, M.D., Commissioner of Food and Drugs, regarding Howard Balin, M.D. (copy attached), the conclusions of this submission as completed on 3/14/75 were re-evaluated. The re-evaluation bears the date of April 1, 1975.

The statistical summary tables and respective probability values as presented in the March 14, 1975 summary of the "8-way" multicenter study were recalculated on the basis of 1569 patients. This excludes

the data of Dr. Balin's 30 patients (Table A-3) from the original 1599 patients. The differences in probability values between the 1569 patient population and 1599 population were minimal and trivial (Tables A-1 and A-2). There is no reason to change any of the overall conclusions of the study.

Inasmuch as the differences were of no consequence, these new and additional tabulations dated 4/1/75 are not intended to replace the prior tabulations of 3/14/75 in the overall summary or in the primary statistical document (Volume 3). These calculations were done only to estimate whether exclusion of Dr. Balin's data would produce any real changes. This was observed not to be true.

Further support showing that exclusion of data from Dr. Balin's patients does not change the data sufficiently to cause a change in the overall conclusion is provided by the attached IBM print-out tabulations. For each print-out the sequence is exclusion, then inclusion, then only the data of Balin, respectively:

#203 Physician's final evaluation (cross tabulation of all patients)--2269 vs. 2308 patients vs. Balin's 39 patients)

#203A Same as above - completed per protocol--(1569 vs. 1599 patients vs. Balin's 30 patients)

#205 Physician's initial evaluation--medication vs. severity of symptoms--(2269 vs. 2308 patients vs. Balin's 39 patients).

Table A-1

Summary Table

Physician's Evaluation

(See footnotes for explanation of table)

Treatment	Effectiveness of Medication: Percentage evaluated moderate or excellent	Effectiveness of Medication: p	Nausea: Percentage improved	Nausea: p	Vomiting: Percentage improved	Vomiting: p
Bendectin	72 (71)	<.01 (<.01)	66 (65)	<.01 (<.01)	79 (77)	.02 (.03)
Doxylamine/ pyridoxine	78 (78)	<.01 (<.01)	76 (75)	<.01 (<.01)	73 (73)	.16 (.17)
Dicyclomine/ doxylamine	78 (78)	<.01 (<.01)	71 (71)	<.01 (<.01)	74 (74)	.07 (.07)
Doxylamine	76 (77)	<.01 (<.01)	71 (69)	<.01 (<.01)	78 (78)	.01 (.01)
Dicyclomine/ pyridoxine	60 (61)	.27 (.28)	58 (57)	.03 (.03)	62 (62)	.64 (.64)
Pyridoxine	66 (66)	.09 (.10)	68 (68)	<.01 (<.01)	67 (66)	.35 (.36)
Dicyclomine	60 (61)	.19 (.17)	63 (61)	.07 (.07)	71 (71)	.32 (.33)
Placebo	56 (57)		53 (52)		66 (66)	

The first numbers are the results of the analysis with Dr. Balin's patients excluded.
The numbers in parentheses are the results of the analysis with Dr. Balin's patients included.
The p values are one-sided probabilities based on tests of each active medication vs. placebo.
The analysis of vomiting includes only those patients with vomiting symptoms at pretreatment.

4/1/75

No 011 592

Table A-2

Summary Table

Patient's Diary Card

(See footnotes for explanation of table)

Treatment	Nausea: Percent reduction from pretreatment	Nausea: p	Vomiting: Percentage with no vomiting on 5 or more treatment days	Vomiting: p
Bendectin	57 (57)	<.01 (<.01)	44 (46)	<.01 (<.01)
Doxylamine/ pyridoxine	65 (64)	<.01 (<.01)	48 (48)	<.01 (<.01)
Dicyclomine/ doxylamine	50 (50)	<.01 (<.01)	49 (49)	<.01 (<.01)
Doxylamine	56 (56)	<.01 (<.01)	55 (54)	<.01 (<.01)
Dicyclomine/ pyridoxine	43 (44)	.02 (.03)	39 (39)	.08 (.08)
Pyridoxine	35 (35)	.07 (.09)	29 (29)	.41 (.37)
Dicyclomine	[illegible] (36)	.25 (.25)	29 (30)	.29 (.26)
Placebo	31 (31)		28 (28)	

The first numbers are the results of the analysis with Dr. Balin's patients excluded.
The numbers in parentheses are the results of the analysis with Dr. Balin's patients included.
The p values are one-sided probabil[illegible] of each active medication vs. placebo.
The analysis of vom[illegible] th vomiting symptoms at pretreatment.

4/1/75

No 011

593

Table A-3

Number of Patients--Balin (Inv. No. 113)

Treatment	No. of Patients
Bendectin	3
Doxylamine/pyridoxine	4
Dicyclomine/doxylamine	3
Doxylamine	4
Dicyclomine/pyridoxine	3
Pyridoxine	3
Dicyclomine	6
Placebo	4
Total	30

4/1/75

203 NAUSEA & VOMITING OF PREGNANCY - COMPARATIVE EFFICACY OF BENDECTIN, EACH OF ITS THREE INGREDIENTS ALONE AND IN COMBINATION
INVESTIGATOR: ALL MINUS BALIN

PERCENTS BASED ON # PTS. ASSIGNED TO EACH DRUG GP	BENTYL #	BENTYL %	DECAPRYN #	DECAPRYN %	BENTYL/ DECAPRYN #	BENTYL/ DECAPRYN %	PYRIDOXINE #	PYRIDOXINE %	BENTYL/ PYRIDOXINE #	BENTYL/ PYRIDOXINE %	DECAPRYN/ PYRIDOXINE #	DECAPRYN/ PYRIDOXINE %	BENDECTIN #	BENDECTIN %	PLACEBO #	PLACEBO %	TOTAL ALL PATIENTS #	TOTAL ALL PATIENTS %
TOTAL PATIENTS: ASSIGNED TO STUDY DRUG	280	104.9	283	105.2	295	105.0	286	106.7	281	106.8	279	106.5	284	108.0	281	106.0	2269	106.1
"NO-RETURNS" EXCLUDED	267		269		281		268		263		262		263		265		2138	
STUDY COMPLETED PER PROTOCOL																		
YES	197	73.8	205	76.2	215	76.5	188	70.1	192	73.0	209	79.8	186	70.7	177	66.8	1569	73.4
NO	83	31.1	78	29.0	80	28.5	98	36.6	89	33.8	70	26.7	98	37.3	104	39.2	700	32.7
NOT STATED																		
REASON NOT COMPLETED																		
PATIENT DID NOT RETURN @	13	4.9	14	5.2	14	5.0	18	6.7	18	6.8	17	6.5	21	8.0	16	6.0	131	6.1
OTHER	70	26.2	64	23.8	66	23.5	80	29.9	71	27.0	53	20.2	77	29.3	88	33.2	569	26.6
SEVERITY OF NAUSEA:																		
NONE	59	22.1	75	27.9	81	28.8	57	21.3	64	24.3	75	28.6	79	30.0	51	19.2	541	25.3
MILD	103	38.6	132	49.1	128	45.6	121	45.1	98	37.3	124	47.3	109	41.4	100	37.7	915	42.8
MODERATE	73	27.3	43	16.0	55	19.6	68	25.4	70	26.6	47	17.9	55	20.9	79	29.8	490	22.9
SEVERE	28	10.5	16	5.9	16	5.7	21	7.8	27	10.3	15	5.7	17	6.5	32	12.1	172	8.0
NOT STATED	4	1.5	3	1.1	1	.4	1	.4	4	1.5	1	.4	3	1.1	3	1.1	20	.9
SEVERITY OF VOMITING:																		
NONE	143	53.6	175	65.1	176	62.6	163	60.8	157	59.7	174	66.4	179	68.1	150	56.6	1317	61.6
MILD	74	27.7	70	26.0	69	24.6	59	22.0	60	22.8	57	21.8	56	21.3	63	23.8	508	23.8
MODERATE	34	12.7	14	5.2	29	10.0	36	13.4	35	13.3	19	7.3	18	6.8	33	12.5	217	10.1
SEVERE	13	4.9	7	2.6	7	2.5	9	3.4	7	2.7	11	4.2	7	2.7	15	5.7	76	3.6
NOT STATED	3	1.1	3	1.1	1	.4	1	.4	4	1.5	1	.4	3	1.1	4	1.5	20	.9
CONCOMITANT RX DURING STUDY																		
NO	85	31.8	91	33.8	90	32.0	93	34.7	85	32.3	82	31.3	96	36.5	106	40.0	728	34.1
YES	178	66.7	174	64.7	188	66.9	173	64.6	175	66.5	177	67.6	165	62.7	157	59.2	1387	64.9
NOT STATED	4	1.5	4	1.5	3	1.1	2	.7	3	1.1	3	1.1	2	.8	2	.8	23	1.1
SIDE EFFECTS VOLUNTEERED																		
NO	236	88.4	224	83.3	240	85.4	240	89.6	228	86.7	237	90.5	223	84.8	[illegible]	88.3	1862	87.1
YES	29	10.9	41	15.2	38	13.5	26	9.7	33	12.5	23	8.8	38	14.4	[illegible]	11.3	258	12.1
NOT STATED	2	.7	4	1.5	3	1.1	2	.7	2	.8	2	.8	2	.8	1	.4	18	.8
EFFECTIVENESS OF MEDICATION:																		
EXCELLENT	80	30.0	103	38.3	103	36.7	78	29.1	75	28.5	115	43.9	101	38.4	62	23.4	717	33.5
MODERATE	65	24.3	88	32.7	103	36.7	77	28.7	67	25.5	80	30.5	76	28.9	68	25.7	624	29.2
SLIGHT	47	[illegible]	39	14.5	39	3.9	47	17.5	47	17.9	30	11.5	39	14.8	54	20.4	342	16.0
NONE	71	26.6	[illegible]	13.4	[illegible]	.4	65	24.3	71	27.0	34	13.0	43	16.3	80	30.2	432	20.2
NOT STATED	4	1.5	3	1.1	4	.4	1	.4	3	1.1	3	1.1	4	1.5	1	.4	23	1.1

@ REMAINING DATA NOT AVAILABLE FOR THESE PATIENTS

203 NAUSEA & VOMITING OF PREGNANCY - COMPARATIVE EFFICACY OF BENDECTIN, EACH OF ITS THREE INGREDIENTS ALONE AND IN COMBINATION
INVESTIGATORS

PERCENTS BASED ON # PTS. ASSIGNED TO EACH DRUG GP	BENTYL #	BENTYL %	DECAPRYN #	DECAPRYN %	BENTYL/ DECAPRYN #	BENTYL/ DECAPRYN %	PYRIDOXINE #	PYRIDOXINE %	BENTYL/ PYRIDOXINE #	BENTYL/ PYRIDOXINE %	DECAPRYN/ PYRIDOXINE #	DECAPRYN/ PYRIDOXINE %	BENDECTIN #	BENDECTIN %	PLACEBO #	PLACEBO %	TOTAL ALL PATIENTS #	TOTAL ALL PATIENTS %
TOTAL PATIENTS: ASSIGNED TO STUDY DRUG	286	104.8	287	105.1	301	105.2	290	106.6	286	106.7	284	106.4	288	107.9	286	105.9	2306	106.1
"NO-RETURNS" EXCLUDED	273		273		286		272		268		267		267		270		2176	
STUDY COMPLETED PER PROTOCOL																		
YES	203	74.4	209	76.6	218	76.2	191	70.2	195	72.8	213	79.8	189	70.8	181	67.0	1599	73.5
NO	83	30.4	78	28.6	83	29.0	99	36.4	91	34.0	71	26.6	99	37.1	105	38.9	709	32.6
NOT STATED																		
REASON NOT COMPLETED																		
PATIENT DID NOT RETURN @	13	4.8	14	5.1	15	5.2	18	6.6	18	6.7	17	6.4	21	7.9	16	5.9	132	6.1
OTHER	70	25.6	64	23.4	68	23.8	81	29.8	73	27.2	54	20.2	78	29.2	89	33.0	577	26.5
SEVERITY OF NAUSEA:																		
NONE	59	21.6	75	27.5	81	28.3	57	21.0	64	23.9	75	28.1	79	29.6	52	19.3	542	24.9
MILD	107	39.2	135	49.5	132	46.2	123	43.2	102	38.1	128	47.9	111	41.6	102	37.8	940	43.2
MODERATE	75	27.5	44	16.1	56	19.6	70	25.7	71	26.5	48	18.0	57	21.3	81	30.0	502	23.1
SEVERE	28	10.3	16	5.9	16	5.6	21	7.7	27	10.1	15	5.6	17	6.4	32	11.9	172	7.9
NOT STATED	4	1.5	3	1.1	1	.3	1	.4	4	1.5	1	.4	3	1.1	3	1.1	20	.9
SEVERITY OF VOMITING:																		
NONE	148	54.2	178	65.2	180	62.9	166	61.0	160	59.7	178	66.7	181	67.8	154	57.0	1345	61.8
MILD	75	27.5	71	26.0	70	24.5	60	22.1	62	23.1	58	21.7	58	21.7	64	23.7	518	23.8
MODERATE	34	12.5	14	5.1	28	9.8	36	13.2	35	13.1	19	7.1	18	6.7	33	12.2	217	10.0
SEVERE	13	4.8	7	2.6	7	2.4	9	3.3	7	2.6	11	4.1	7	2.6	15	5.6	76	3.5
NOT STATED	3	1.1	3	1.1	1	.3	1	.4	4	1.5	1	.4	3	1.1	4	1.5	20	.9
CONCOMITANT RX DURING STUDY																		
NO	90	33.0	95	34.8	94	32.9	97	35.7	90	33.6	86	32.2	99	37.1	110	40.7	761	35.0
YES	179	65.6	174	63.7	189	66.1	173	63.6	175	65.3	178	66.7	166	62.2	158	58.5	1392	64.0
NOT STATED	4	1.5	4	1.5	3	1.0	2	.7	3	1.1	3	1.1	2	.7	2	.7	23	1.1
SIDE EFFECTS VOLUNTEERED																		
NO	242	88.6	228	83.5	245	85.7	244	89.7	233	86.9	242	90.6	227	85.0	239	88.5	1900	87.3
YES	29	10.6	41	15.0	38	13.3	26	9.6	33	12.3	23	8.6	38	14.2	30	11.1	258	11.9
NOT STATED	2	.7	4	1.5	3	1.0	2	.7	2	.7	2	.7	2	.7	1	.4	18	.8
EFFECTIVENESS OF MEDICATION:																		
EXCELLENT	84	30.8	105	39.5	107	37.4	81	29.8	77	28.7	117	43.8	103	38.6	64	23.7	738	33.9
MODERATE	66	24.2	90	33.0	104	36.4	77	28.3	70	26.1	81	30.3	76	28.5	71	26.3	635	29.2
SLIGHT	48	17.5	39	14.3	39	13.5	47	17.3	47	17.5	31	11.6	41	15.4	54	20.0	346	15.9
NONE	71	26.0	36	13.2	32	11.2	66	24.3	71	26.5	35	13.1	43	16.1	80	29.6	434	19.9
NOT STATED	4	1.5	3	1.1	4	1.4	1	.4	3	1.1	3	1.1	4	1.5	1	.4	23	1.1

@ REMAINING DATA NOT AVAILABLE FOR THESE PATIENTS

12/03/74 PAGE 6

203 NAUSEA & VOMITING OF PREGNANCY - COMPARATIVE EFFICACY OF BENDECTIN, EACH OF ITS THREE INGREDIENTS ALONE AND IN COMBINATION

INVESTIGATOR: BALIN

PERCENTS BASED ON # PTS. ASSIGNED TO EACH DRUG OF TOTAL PATIENTS:	BENTYL # %	DECAPRYN # %	BENTYL/ DECAPRYN # %	PYRIDOXINE # %	BENTYL/ PYRIDOXINE # %	DECAPRYN/ PYRIDOXINE # %	BENDECTIN # %	PLACEBO # %	TOTAL ALL PATIENTS # %
ASSIGNED TO STUDY DRUG	6 100.0	4 100.0	6 120.0	4 100.0	5 100.0	5 100.0	4 100.0	5 100.0	39 102.6
"NO-RETURNS" EXCLUDED	6	4	5	4	5	5	4	5	38
**STUDY COMPLETED PER PROTOCOL**									
YES	6 100.0	4 100.0	3 60.0	3 75.0	3 60.0	4 80.0	3 75.0	4 80.0	30 78.9
NO			3 40.0	1 25.0	2 40.0	1 20.0	1 25.0	1 20.0	9 23.7
NOT STATED									
**REASON NOT COMPLETED**									
PATIENT DID NOT RETURN@			1 20.0						1 2.6
OTHER			2 40.0	1 25.0	2 40.0	1 20.0	1 25.0	1 20.0	8 21.1
**SEVERITY OF NAUSEA**									
NONE								1 20.0	1 2.6
MILD	4 66.7	3 75.0	4 80.0	2 50.0	4 80.0	4 80.0	2 50.0	2 40.0	25 65.8
MODERATE	2 33.3	1 25.0	1 20.0	2 50.0	1 20.0	1 20.0	2 50.0	2 40.0	12 31.6
SEVERE									
NOT STATED									
**SEVERITY OF VOMITING**									
NONE	5 83.3	3 75.0	4 80.0	3 75.0	3 60.0	4 80.0	2 50.0	4 80.0	28 73.7
MILD	1 16.7	1 25.0	1 20.0	1 25.0	2 40.0	1 20.0	2 50.0	1 20.0	10 26.3
MODERATE									
SEVERE									
NOT STATED									
**CONCOMITANT RX DURING STUDY**									
NO	5 83.3	4 100.0	4 80.0	4 100.0	5 100.0	4 80.0	3 75.0	4 80.0	33 86.8
YES	1 16.7		1 20.0			1 20.0	1 25.0	1 20.0	5 13.2
NOT STATED									
**SIDE EFFECTS VOLUNTEERED**									
NO	6 100.0	4 100.0	5 100.0	4 100.0	5 100.0	5 100.0	4 100.0	5 100.0	38 100.0
YES									
NOT STATED									
**EFFECTIVENESS OF MEDICATION**									
EXCELLENT	4 66.7	2 50.0	4 80.0	3 75.0	2 40.0	2 40.0	2 50.0	2 40.0	21 55.3
MODERATE	1 16.7	2 50.0	1 20.0		3 60.0	1 20.0		3 60.0	11 28.9
SLIGHT	1 16.7					1 20.0	2 50.0		4 10.5
NONE				1 25.0		1 20.0			2 5.3
NOT STATED									

@ REMAINING DATA NOT AVAILABLE FOR THESE PATIENTS

No 011

5.97

011

03/27/75 PAGE 29

2031 NAUSEA & VOMITING OF PREGNANCY - COMPARATIVE EFFICACY OF BENDECTIN, EACH OF ITS THREE INGREDIENTS ALONE AND IN COMBINATION
INVESTIGATOR: ALL MINUS BALIN

PERCENTS BASED ON # PTS. ASSIGNED TO EACH DRUG GP	BENTYL* #	%	DECAPRYN #	%	BENTYL/ DECAPRYN #	%	PYRIDOXINE #	%	BENTYL/ PYRIDOXINE #	%	DECAPRYN/ PYRIDOXINE #	%	BENDECTIN #	%	PLACEBO #	%	TOTAL ALL PATIENTS #	%
TOTAL PATIENTS: ASSIGNED TO STUDY DRUG	197	100.0	205	100.0	215	100.0	188	100.0	192	100.0	209	100.0	186	100.0	177	100.0	1569	100.0
"NO-RETURNS" EXCLUDED	197		205		215		188		192		209		186		177		1569	
STUDY COMPLETED PER PROTOCOL																		
YES	197	100.0	205	100.0	215	100.0	188	100.0	192	100.0	209	100.0	186	100.0	177	100.0	1569	100.0
NO																		
NOT STATED																		
REASON NOT COMPLETED																		
PATIENT DID NOT RETURN&																		
OTHER																		
SEVERITY OF NAUSEA:																		
NONE	40	20.3	59	28.8	62	28.8	43	22.9	47	24.5	58	27.8	52	28.0	33	18.6	394	25.1
MILD	84	42.6	103	50.2	102	47.4	87	46.3	80	41.7	104	49.8	83	44.6	71	40.1	714	45.5
MODERATE	60	30.5	32	15.6	43	20.0	51	27.1	52	27.1	39	18.7	45	24.2	58	32.8	380	24.2
SEVERE	12	6.1	11	5.4	8	3.7	7	3.7	13	6.8	8	3.8	6	3.2	14	7.9	79	5.0
NOT STATED	1	.5													1	.6	2	.1
SEVERITY OF VOMITING:																		
NONE	114	57.9	132	64.4	135	62.8	119	63.3	119	62.0	135	64.6	132	71.0	106	59.9	992	63.2
MILD	55	27.9	61	29.8	56	26.0	43	22.9	45	23.4	51	24.4	40	21.5	45	25.4	396	25.2
MODERATE	23	11.7	10	4.9	20	9.3	23	12.2	25	13.0	18	8.6	14	7.5	19	10.7	152	9.7
SEVERE	4	2.0	2	1.0	4	1.9	3	1.5	3	1.6	5	2.4			5	2.8	26	1.7
NOT STATED	1	.5													2	1.1	3	.2
CONCOMITANT RX DURING STUDY																		
NO	59	29.9	69	33.7	71	33.0	66	35.1	60	31.3	68	32.5	69	37.1	78	44.1	540	34.4
YES	137	69.5	135	65.9	143	66.5	122	64.9	132	68.8	141	67.5	117	62.9	98	55.4	1025	65.3
NOT STATED	1	.5	1	.5	1	.5									1	.6	4	.3
SIDE EFFECTS VOLUNTEERED																		
NO	183	92.9	177	86.3	187	87.0	171	91.0	171	89.1	197	94.3	158	84.9	161	91.0	1405	89.5
YES	14	7.1	28	13.7	26	12.1	17	9.0	21	10.9	11	5.3	28	15.1	16	9.0	161	10.3
NOT STATED					2	.9					1	.5					3	.2
EFFECTIVENESS OF MEDICATION:																		
EXCELLENT	64	32.5	65	41.5	82	38.1	58	30.9	59	30.7	95	45.5	74	39.8	47	26.6	564	35.9
MODERATE	55	27.9	71	34.6	84	39.1	66	35.1	57	29.7	68	32.5	59	31.7	52	29.4	512	32.6
SLIGHT	38	19.3	32	15.6	32	14.9	29	15.4	35	18.2	27	12.9	32	17.2	43	24.3	268	17.1
NONE	40	20.3	17	8.3	16	7.4	35	18.6	41	21.4	19	9.1	20	10.8	35	19.8	223	14.2
NOT STATED					1	.5							[illegible]	.5			2	.1

& REMAINING DATA NOT AVAILABLE FOR THESE PATIENTS

203A NAUSEA & VOMITING OF PREGNANCY - COMPARATIVE EFFICACY OF BENDECTIN, EACH OF ITS THREE INGREDIENTS ALONE AND IN COMBINATION

INVESTIGATOR:

PERCENTS BASED ON # PTS. ASSIGNED TO EACH DRUG GP	BENTYL #	BENTYL %	DECAPRYN #	DECAPRYN %	BENTYL/ DECAPRYN #	BENTYL/ DECAPRYN %	PYRIDOXINE #	PYRIDOXINE %	BENTYL/ PYRIDOXINE #	BENTYL/ PYRIDOXINE %	DECAPRYN/ PYRIDOXINE #	DECAPRYN/ PYRIDOXINE %	BENDECTIN #	BENDECTIN %	PLACEBO #	PLACEBO %	TOTAL ALL PATIENTS #	TOTAL ALL PATIENTS %
TOTAL PATIENTS:																		
ASSIGNED TO STUDY DRUG	203	100.0	209	100.0	218	100.0	191	100.0	195	100.0	213	100.0	139	100.0	181	100.0	1599	100.0
"NO-RETURNS" EXCLUDED	203		209		218		191		195		213		139		181		1599	
STUDY COMPLETED PER PROTOCOL																		
YES	203	100.0	209	100.0	218	100.0	191	100.0	195	100.0	213	100.0	139	100.0	181	100.0	1599	0.0
NO																		
NOT STATED																		
REASON NOT COMPLETED																		
PATIENT DID NOT RETURN																		
OTHER																		
SEVERITY OF NAUSEA																		
NONE	40	19.7	59	28.2	62	28.4	43	22.5	47	24.1	58	27.2	52	27.5	33	18.2	394	24.6
MILD	88	43.3	106	50.7	105	48.2	89	46.6	82	42.1	107	50.2	84	44.4	73	40.3	734	45.9
MODERATE	62	30.5	33	15.8	43	19.7	52	27.2	53	27.2	40	18.8	47	24.9	60	33.1	390	24.4
SEVERE	12	5.9	11	5.3	8	3.7	7	3.7	13	6.7	8	3.8	6	3.2	14	7.7	79	4.9
NOT STATED	1	.5													1	.6	2	.1
SEVERITY OF VOMITING:																		
NONE	119	58.6	135	64.5	137	62.8	121	63.4	120	61.5	138	64.8	133	70.4	109	60.2	1012	63.3
MILD	56	27.6	62	29.7	57	26.1	44	23.0	47	24.1	52	24.4	42	22.2	46	25.4	405	25.4
MODERATE	23	11.3	10	4.8	20	9.2	23	12.0	25	12.8	18	9.5	14	7.4	19	10.5	152	9.5
SEVERE	4	2.0	2	1.0	4	1.8	3	1.5	3	1.5	5	2.3			5	2.8	26	1.6
NOT STATED	1	.5													2	1.1	3	.2
CONCOMITANT RX DURING STUDY																		
NO	64	31.5	73	34.9	74	33.9	69	36.1	63	32.3	72	33.8	72	38.1	82	45.3	569	35.6
YES	138	68.0	135	64.6	143	65.6	122	63.9	132	67.7	141	66.2	117	61.9	98	54.1	1026	64.2
NOT STATED	1	.5	1	.5	1	.5									1	.6	4	.3
SIDE EFFECTS VOLUNTEERED																		
NO	189	93.1	181	86.6	190	87.2	174	91.1	174	89.2	201	94.4	161	85.2	165	91.2	1435	89.7
YES	14	6.9	28	13.4	26	11.9	17	8.9	21	10.8	11	5.2	28	14.8	16	8.8	161	10.1
NOT STATED					2	.9					1	.5					3	.2
EFFECTIVENESS OF MEDICATION:																		
EXCELLENT	68	33.5	87	41.6	85	39.0	60	31.4	60	30.8	97	45.5	75	39.7	48	26.5	580	36.3
MODERATE	56	27.6	73	34.9	84	38.5	66	34.6	59	30.3	68	31.9	59	31.2	55	30.4	520	32.5
SLIGHT	39	19.2	32	15.3	32	14.7	29	15.2	35	17.9	28	13.1	34	18.0	43	23.8	272	17.0
NONE	40	19.7	17	8.1	16	7.3	36	18.8	41	21.0	20	9.4	20	10.6	35	19.3	225	14.1
NOT STATED					1	.5							1	.5			2	.1

No 011

203 NAUSEA & VOMITING OF PREGNANCY – COMPARATIVE EFFICACY OF BENDECTIN, EACH OF ITS THREE INGREDIENTS ALONE AND IN COMBINATION

INVESTIGATOR:
DATA:

PERCENTS BASED ON # PTS. ASSIGNED TO EACH DRUG GP	BENTYL # %	DECAPRYN # %	BENTYL/ DECAPRYN # %	PYRIDOXINE # %	BENTYL/ PYRIDOXINE # %	DECAPRYN/ PYRIDOXINE # %	BENDECTIN # %	PLACEBO # %	TOTAL ALL PATIENTS # %
TOTAL PATIENTS:									
ASSIGNED TO STUDY DRUG	6 100.0	4 100.0	3 100.0	3 100.0	3 100.0	4 100.0	3 100.0	4 100.0	30 100.0
"NO-RETURNS" EXCLUDED	6	4	3	3	3	4	3	4	30
STUDY COMPLETED PER PROTOCOL									
YES	6 100.0	4 100.0	3 100.0	3 100.0	3 100.0	4 100.0	3 100.0	4 100.0	30 100.0
NO									
NOT STATED									
REASON NOT COMPLETED									
PATIENT DID NOT RETURN									
OTHER									
SEVERITY OF NAUSEA:									
NONE									
MILD	4 66.7	3 75.0	3 100.0	2 66.7	2 66.7	3 75.0	1 33.3	2 50.0	20 66.7
MODERATE	2 33.3	1 25.0		1 33.3	1 33.3	1 25.0	2 66.7	2 50.0	10 33.3
SEVERE									
NOT STATED									
SEVERITY OF VOMITING:									
NONE	5 83.3	3 75.0	2 66.7	2 66.7	1 33.3	3 75.0	1 33.3	3 75.0	20 66.7
MILD	1 16.7	1 25.0	1 33.3	1 33.3	2 66.7	1 25.0	2 66.7	1 25.0	10 33.3
MODERATE									
SEVERE									
NOT STATED									
CONCOMITANT RX DURING STUDY									
NO	5 83.3	4 100.0	3 100.0	3 100.0	3 100.0	4 100.0	3 100.0	4 100.0	29 96.7
YES	1 16.7								1 3.3
NOT STATED									
SIDE EFFECTS VOLUNTEERED									
NO	6 100.0	4 100.0	3 100.0	3 100.0	3 100.0	4 100.0	3 100.0	4 100.0	30 100.0
YES									
NOT STATED									
EFFECTIVENESS OF MEDICATION:									
EXCELLENT	4 66.7	2 50.0	3 100.0	2 66.7	1 33.3	2 50.0	1 33.3	1 25.0	15 53.3
MODERATE	1 16.7	2 50.0			2 66.7			3 75.0	[illegible] 26.7
SLIGHT	1 16.7					1 25.0	2 66.7		4 13.3
NONE				1 33.3		1 25.0			2 6.7
NOT STATED									

* REMAINING DATA NOT AVAILABLE FOR THESE PATIENTS

No 011

600

205 NAUSEA & VOMITING OF PREGNANCY – COMPARATIVE EFFICACY OF BENDECTIN, EACH OF ITS INGREDIENTS ALONE AND IN COMBINATION

INVESTIGATOR: ALL MINUS BALIN

GRAVID: ALL

I N I T I A L   E V A L U A T I O N

	BENTYL		DECAPRYN		BENTYL/ DECAPRYN		PYRIDOXINE		BENTYL / PYRIDOXINE		DECAPRYN/ PYRIDOXINE		BENDECTIN		PLACEBO		TOTAL ALL PATIENTS	
SEVERITY OF NAUSEA																		
NONE	1	.4					1	.3					1	.4			3	.1
MILD	60	21.4	66	23.3	55	18.6	55	19.2	66	23.5	50	17.9	67	23.6	64	22.8	483	21.3
MODERATE	141	50.4	153	54.1	180	61.0	150	52.4	147	52.3	147	52.7	154	54.2	143	50.9	1215	53.5
SEVERE	77	27.5	64	22.6	59	20.0	80	28.0	68	24.2	81	29.0	61	[illegible]	74	26.3	564	24.9
NOT STATED	1	.4			1	.3					1	.4	1	.4			4	.2
DURATION OF NAUSEA *																		
1 - 2 DAYS	12	4.3	7	2.5	6	2.0	5	1.7	10	3.6	6	2.2	10	3.5	6	2.1	62	2.7
3 - 7	58	20.7	62	21.9	56	19.0	58	20.3	62	22.1	58	20.8	60	21.1	57	20.3	471	20.8
8 - 14	77	27.5	63	22.3	86	29.2	79	27.6	72	25.6	81	29.0	84	29.6	77	27.4	619	27.3
> 14	132	47.1	148	52.3	146	49.5	142	49.7	136	48.4	132	47.3	126	44.4	140	49.8	1102	48.6
NOT STATED			3	1.1	1	.3	1	[illegible]	1	.4	2	.7	3	1.1	1	.4	12	.5
SEVERITY OF VOMITING																		
NONE	131	46.8	124	43.8	106	35.9	124	43.4	130	46.3	122	43.7	133	46.8	104	37.0	974	42.9
MILD	64	22.9	83	29.3	80	27.1	67	23.4	81	28.8	71	25.4	75	28.4	88	31.3	609	26.8
MODERATE	62	22.1	55	19.4	79	26.8	66	23.1	52	18.5	59	21.1	56	19.7	64	22.8	493	21.7
SEVERE	22	7.9	20	7.1	30	10.2	29	10.1	18	6.4	26	9.3	20	7.0	25	8.9	190	8.4
NOT STATED	1	.4	1	.4							1	.4					3	.1
DURATION OF VOMITING *																		
1 - 2 DAYS	22	7.5	29	10.2	41	13.9	28	9.8	29	10.3	22	7.9	32	11.3	22	7.8	225	9.9
3 - 7	32	11.4	39	13.8	41	13.9	43	15.0	43	15.3	37	13.3	32	11.3	56	19.9	323	14.2
8 - 14	39	13.9	35	12.4	37	12.5	34	11.9	33	11.7	29	10.4	39	13.7	34	12.1	280	12.3
> 14	56	20.0	54	19.1	69	23.4	56	19.6	45	16.0	69	24.7	48	16.9	65	23.1	462	20.4
NOT STATED			2	.7	1	.3	1	.3	1	.4							5	.2
TOTAL	280	100.0	283	100.0	295	100.0	286	100.0	281	100.0	279	100.0	284	100.0	281	100.0	2269	100.0

* PATIENTS WHO HAD NO NAUSEA OR NO VOMITING DO NOT REFLECT A SCORE FOR DURATION.

601

022

08/19/74 PAGE 30

205 NAUSEA & VOMITING OF PREGNANCY - COMPARATIVE EFFICACY OF BENDECTIN, EACH OF ITS INGREDIENTS ALONE AND IN COMBINATION
INVESTIGATOR:

GRAVIDA ALL

INITIAL EVALUATION

	BENTYL		DECAPRYN		BENTYL/ DECAPRYN		PYRIDOXINE		BENTYL / PYRIDOXINE		DECAPRYN/ PYRIDOXINE		BENDECTIN		PLACEBO		TOTAL ALL PATIENTS	
SEVERITY OF NAUSEA																		
NONE	1	.3					1	.3					1	.3			3	.1
MILD	61	22.0	69	24.0	56	18.6	55	19.0	69	24.1	52	18.3	69	24.0	67	23.4	500	21.7
MODERATE	144	50.3	154	53.7	185	61.5	154	53.1	149	52.1	150	52.8	156	54.2	145	50.7	1237	53.6
SEVERE	77	26.9	64	22.3	59	19.6	80	27.6	68	23.8	81	28.5	61	21.2	74	25.9	564	24.4
NOT STATED	1	.3			1	.3					1	.4	1	.3			4	.2
DURATION OF NAUSEA *																		
1 - 2 DAYS	13	4.5	8	2.8	7	2.3	5	1.7	11	3.8	8	2.8	11	3.8	8	2.8	71	3.1
3 - 7	63	22.0	64	22.3	59	19.6	61	21.0	65	22.7	61	21.5	62	21.5	58	20.3	493	21.4
8 - 14	77	26.9	64	22.3	88	29.2	80	27.6	73	25.5	81	28.5	85	29.5	78	27.3	626	27.1
> 14	132	46.2	148	51.6	146	48.5	142	49.0	136	47.6	132	46.5	126	43.8	141	49.3	1103	47.8
NOT STATED			3	1.0	1	.3	1	.3	1	.3	2	.7	3	1.0	1	.3	12	.5
SEVERITY OF VOMITING																		
NONE	136	47.6	127	44.3	112	37.2	127	43.8	135	47.2	126	44.4	135	46.9	109	38.1	1007	43.6
MILD	65	22.7	84	29.3	80	26.6	68	23.4	81	28.3	72	25.4	77	26.7	88	30.8	615	26.6
MODERATE	62	21.7	55	19.2	79	26.2	66	22.8	52	18.2	59	20.8	56	19.4	64	22.4	493	21.4
SEVERE	22	7.7	20	7.0	30	10.0	29	10.0	18	6.3	26	9.2	20	6.9	25	8.7	190	8.2
NOT STATED	1	.3	1	.3							1	.4					3	.1
DURATION OF VOMITING *																		
1 - 2 DAYS	23	8.0	30	10.5	41	13.6	29	10.0	29	10.1	23	8.1	34	11.8	22	7.7	231	10.0
3 - 7	32	11.2	39	13.6	41	13.6	43	14.8	43	15.0	37	13.0	32	11.1	56	19.6	323	14.0
8 - 14	39	13.6	35	12.2	37	12.3	34	11.7	33	11.5	29	10.2	39	13.5	34	11.9	280	12.1
> 14	56	19.6	54	18.8	69	22.9	56	19.3	45	15.7	69	24.3	48	16.7	65	22.7	462	20.0
NOT STATED			2	.7	1	.3	1	.3	1	.3							5	.2
TOTAL	286	100.0	287	100.0	301	100.0	290	100.0	286	100.0	284	100.0	288	100.0	286	100.0	2308	100.0

* PATIENTS WHO HAD NO NAUSEA OR NO VOMITING DO NOT REFLECT A SCORE FOR DURATION.

08/19/74 PAGE 6

205 NAUSEA & VOMITING OF PREGNANCY - COMPARATIVE EFFICACY OF BENDECTIN, EACH OF ITS INGREDIENTS ALONE AND IN COMBINATION

INVESTIGATOR:
BALIN
GRAVIDS ALL

INITIAL EVALUATION

	BENTYL		DECAPRYN		BENTYL/ DECAPRYN		PYRIDOXINE		BENTYL / PYRIDOXINE		DECAPRYN/ PYRIDOXINE		BENDECTIN		PLACEBO		TOTAL ALL PATIENTS	
SEVERITY OF NAUSEA																		
NONE																		
MILD	3	50.0	3	75.0	1	16.7			3	60.0	2	40.0	2	50.0	3	60.0	17	43.6
MODERATE	3	50.0	1	25.0	5	83.3	4	100.0	2	40.0	3	60.0	2	50.0	2	40.0	22	56.4
SEVERE																		
NOT STATED																		
DURATION OF NAUSEA *																		
1 - 2 DAYS	1	16.7	1	25.0	1	16.7			1	20.0	2	40.0	1	25.0	2	40.0	9	23.1
3 - 7	5	83.3	2	50.0	3	50.0	3	75.0	3	60.0	3	60.0	2	50.0	1	20.0	22	56.4
8 - 14			1	25.0	2	33.3	1	25.0	1	20.0			1	25.0	1	20.0	7	17.9
> 14															1	20.0	1	2.6
NOT STATED																		
SEVERITY OF VOMITING																		
NONE	5	83.3	3	75.0	6	100.0	3	75.0	5	100.0	4	80.0	2	50.0	5	100.0	33	84.6
MILD	1	16.7	1	25.0			1	25.0			1	20.0	2	50.0			6	15.4
MODERATE																		
SEVERE																		
NOT STATED																		
DURATION OF VOMITING *																		
1 - 2 DAYS	1	16.7	1	25.0			1	25.0			1	20.0	2	50.0			6	15.4
3 - 7																		
8 - 14																		
> 14																		
NOT STATED																		
TOTAL	6	100.0	4	100.0	6	100.0	4	100.0	5	100.0	5	100.0	4	100.0	5	100.0	39	100.0

* PATIENTS WHO HAD NO NAUSEA OR NO VOMITING DO NOT REFLECT A SCORE FOR DURATION.

Protocol 008- -004

NAUSEA AND VOMITING OF PREGNANCY
COMPARATIVE EFFICACY OF BENDECTIN,® EACH OF ITS THREE INGREDIENTS ALONE AND IN COMBINATION, AND PLACEBO

INVESTIGATOR

OBJECTIVE

To evaluate the relative therapeutic efficacy of Bendectin®, each of its 3 ingredients alone and in combination, and placebo in the management of nausea and/or vomiting of the first trimester of pregnancy.

PATIENTS

Forty or more patients complaining of nausea and/or vomiting and who are in the first trimester of pregnancy (first 12 weeks of gestation) will be admitted to the study by each primary investigator. This will be a multi-clinic study involving more than 14 clinics. No patient will be given more than one of the 8 medications.

Only those patients who, in the opinion of the investigator, will be co-operative and complete the questionnaires will be included in the study.

Patient consent will be obtained by the investigator.

606.

No 0 1 1

MEDICATIONS

All medication will be identical in appearance and all will have the same special coating now used for Bendectin.

1. Dicyclomine. Each tablet will contain 10 mg. dicyclomine hydrochloride (Bentyl®).
2. Doxylamine. Each tablet will contain 10 mg. doxylamine succinate (Decapryn®).
3. Dicyclomine/doxylamine combination (10 mg. each of above).
4. Placebo.
5. Pyridoxine. Each tablet will contain 10 mg. of pyridoxine hydrochloride.
6. Dicyclomine/pyridoxine. Each tablet will contain 10 mg. each of pyridoxine hydrochloride and dicyclomine hydrochloride.
7. Doxylamine/pyridoxine. Each tablet will contain 10 mg. each of pyridoxine hydrochloride and doxylamine succinate.
8. Bendectin®. Each tablet will contain 10 mg. each of dicyclomine hydrochloride, doxylamine succinate, and pyridoxine hydrochloride.

Each medication will be packaged in bottles of 30 tablets and each bottle will be labeled with a Chinese tear-off label for blinding purposes. The sealed tear-off portion will contain the identity of the contents. The portion remaining on the bottle will have the patient number. (The Chinese tear-off label will be opened only in case of a serious problem and after contact with the project monitor at Merrell-National Laboratories.)

PROCEDURE

This will be a double-blind study, in which distribution of medication

will be randomized. At the initial visit, each patient will be given a pretreatment "In Dr.'s Office" daily diary card to complete and leave with the investigator. The investigator will complete the initial evaluation, physician's report form at the initial visit, recording name, age, dates, LMP, and supplying all information requested.

Each patient, in sequence, will then be given a bottle of medication. The tear-off portion of the label will be removed, attached to the physician's initial evaluation report form, and returned unopened to Merrell-National Laboratories when the patient has completed the study. Each patient will be instructed to take 2 tablets at bedtime for 7 nights and if necessary, 1 additional tablet in the mornings and/or in the midafternoons.

Each patient will be given 7 daily report forms to be completed by her at home. The importance of answering all questions and completing the form <u>each day</u>, not waiting until the end of the study to complete all forms at once, shall be especially emphasized.

At the end of 7 days of treatment, each patient will return to the investigator and return all daily diary cards to him/her. The investigator will check the forms (including the pretreatment form) for completeness and will obtain any omitted or needed information before the patient leaves. At this visit, the investigator will complete the final evaluation, physician's report form. The FDA-required separate Drug Experience Report form (FD-1639) will be completed for every patient who experiences serious side effects.

[illegible] completed report forms (including pretreatment and post-treatment daily diary forms) for each patient will be submitted to the Project Monitor at Merrell-National Laboratories at about 1- or 2-week intervals.

A final evaluation will be submitted on each patient entered into the study. "Study completed according to protocol" is defined as availability of 7 or 8 patient diary cards (including "In Dr.'s Office" card) with one or more check marks in each part of each diary card. The reasons for discontinuance of therapy and/or patient "dropouts" will be clearly stated.

Close liaison will be maintained between the investigator and the Merrell-National Project Monitor. Since all supplies of an investigative drug are strictly accountable to the FDA, the Merrell-National Laboratories will reserve the right to recall the medication and terminate the study at any time prior to completion of the study period, now anticipated to be less than 1 year.

EVALUATION OF RESULTS AND STATISTICAL ANALYSIS

Therapeutic efficacy will be measured by: 1) hours of nausea and frequency of vomiting reported on the patients' daily diary cards among the 8 medication groups; and 2) one or more of the judgment factors (e.g. overall efficacy of medication) reported by the physicians. The data will be subjected to a statistical analysis by the Biostatistical Department of Merrell-National Laboratories.

REPORT FORMS

The following report forms will be supplied by Merrell-National: Physician's Initial Evaluation, Physician's Final Evaluation, and Patient's Daily Diary Card.

_ M.D.

Date

No Carbon Required - Do Not Place On Other NCR Forms While Filling In 0 1 1 610

Do Not Write In This Space

NAUSEA AND VOMITING OF PREGNANCY
PHYSICIAN'S REPORT FORM
INITIAL EVALUATION

PROTOCOL NO.:

PATIENT'S NAME: AGE:

PLEASE ATTACH TEAR-OFF PORTION OF APPROPRIATE MEDICATION LABEL HERE.

MARITAL STATUS: ☐ MARRIED ☐ SINGLE ☐ OTHER

RACE: ☐ CAUC. ☐ NEGRO ☐ ORIENT. ☐ AM. IND. ☐ OTHER

MEDICATION NUMBER

GRAVIDA: (INCLUDING THIS PREGNANCY)

PARA: ABORTUS: ____

INITIAL EVALUATION: (PRETREATMENT) ON DATE OF ENTERING STUDY

TODAY'S DATE: MONTH DAY YEAR

START OF LMP: MONTH DAY YEAR

ESTIMATED DURATION OF GESTATION: (WEEKS, AS BASED ON ESTIMATED DATE OF CONCEPTION)

TODAY'S WEIGHT: (LB.)

RECENT HISTORY OF PATIENT'S NAUSEA AND VOMITING OF PREGNANCY:

NAUSEA: ☐ NONE, VOMITING ONLY ☐ YES IF YES, CHECK BOTH SEVERITY AND DURATION.

SEVERITY OF NAUSEA: ☐ MILD ☐ MODERATE ☐ SEVERE

DURATION OF NAUSEA: ☐ 1-2 DAYS ☐ 3-7 DAYS ☐ 8-14 DAYS ☐ MORE THAN 14 DAYS

VOMITING: ☐ NONE, NAUSEA ONLY ☐ YES IF YES, CHECK BOTH SEVERITY AND DURATION.

SEVERITY OF VOMITING: ☐ MILD ☐ MODERATE ☐ SEVERE

DURATION OF VOMITING: ☐ 1-2 DAYS ☐ 3-7 DAYS ☐ 8-14 DAYS ☐ MORE THAN 14 DAYS

NAUSEA AND VOMITING OF PREGNANCY

PHYSICIAN'S REPORT FORM

PROTOCOL NO.:

FINAL EVALUATION

PATIENT'S NAME: AGE: MEDICATION NUMBER:

FINAL EVALUATION: (TO BE COMPLETED AT END OF STUDY)

DATE OF PATIENT'S VISIT: MONTH: DAY: YEAR:

STUDY COMPLETED ACCORDING TO PROTOCOL CRITERIA?

☐ YES ☐ NO - IF NO, GIVE REASON: ☐ PATIENT DID NOT RETURN

☐ OTHER (SPECIFY)

TODAY'S WEIGHT: (LB.)

SEVERITY OF PATIENT'S NAUSEA: ☐ NONE ☐ MILD ☐ MODERATE ☐ SEVERE

SEVERITY OF PATIENT'S VOMITING: ☐ NONE ☐ MILD ☐ MODERATE ☐ SEVERE

HAS PATIENT TAKEN ANY OTHER MEDICATION DURING THIS TIME? ☐ NO ☐ YES (IF YES, SPECIFY)

	NAME	DOSE/DAY	DATES OF MEDICATION
☐ VITAMINS (SPECIFY)			
☐ IRON (SPECIFY)			
☐ HYPNOTIC (SPECIFY)			
☐ ANALGESIC (SPECIFY)			
☐ ANTIHISTAMINE (SPECIFY)			
☐ OTHER (SPECIFY)			

WERE ANY SIDE EFFECTS VOLUNTEERED BY PATIENT? ☐ NO ☐ YES (LIST BELOW)

EFFECTIVENESS OF MEDICATION: ☐ EXCELLENT ☐ MODERATE ☐ SLIGHT ☐ NONE

OVERALL EVALUATION AND COMMENTS:

DO NOT WRITE IN THIS SPACE

612

PATIENT'S DAILY DIARY CARD

PATIENT'S NAME:

DR.'S NAME:

STUDY DAY (CHECK ONE)

☐ IN DR.'S OFFICE BEFORE START OF MEDICATION. (DO NOT FILL OUT PART C TODAY) (REMEMBER TO FILL OUT DAY-1 CARD TOMORROW EVENING)

☐ DAY 1 (DAY AFTER SEEING YOUR DOCTOR)

☐ DAY 2 ☐ DAY 3 ☐ DAY 4 ☐ DAY 5 ☐ DAY 6 ☐ DAY 7

PART A: NAUSEA

NAUSEA PRESENT ON THIS DAY? ☐ YES (IF YES, CHECK 1 BOX IN EACH OF THE 3 COLUMNS) ☐ NO (IF NO, GO DIRECTLY TO PART B)

1. DURATION
   - ☐ LESS THAN 1 HOUR
   - ☐ 1 TO 2 HOURS
   - ☐ 3 TO 4 HOURS
   - ☐ 5 TO 6 HOURS
   - ☐ 7 TO 12 HOURS
   - ☐ 13 TO 16 HOURS
   - ☐ 17 TO 24 HOURS
2. EFFECT ON USUAL DAILY ACTIVITIES
   - ☐ NONE
   - ☐ SOMEWHAT REDUCED
   - ☐ MUCH REDUCED
   - ☐ UNABLE TO PERFORM ANY USUAL DAILY ACTIVITIES
3. EFFECT ON EATING
   - ☐ NONE
   - ☐ ATE SOMEWHAT LESS
   - ☐ ATE MUCH LESS
   - ☐ UNABLE TO EAT ANYTHING AT ALL

(FOLD HERE)

PART B: VOMITING

VOMITING PRESENT ON THIS DAY? ☐ YES (IF YES, CHECK 1 BOX IN EACH OF THE 2 COLUMNS) ☐ NO (IF NO, GO DIRECTLY TO PART C)

1. TYPE OF VOMITING
   - ☐ GAGGING ONLY
   - ☐ DRY HEAVING
   - ☐ SOME VOMITING
   - ☐ HEAVY VOMITING
2. FREQUENCY
   - ☐ 1 TIME THIS DAY
   - ☐ 2 TIMES THIS DAY
   - ☐ 3-5 TIMES THIS DAY
   - ☐ 6-12 TIMES THIS DAY
   - ☐ MORE THAN 12 TIMES THIS DAY

PART C: MEDICINE FOR NAUSEA AND VOMITING DURING PAST 24 HOURS (CHECK EACH TIME THE MEDICINE WAS TAKEN)

☐ 2 TABLETS LAST NIGHT BEFORE GOING TO BED ☐ 1 TABLET THIS MORNING ☐ 1 TABLET THIS AFTERNOON ☐ NONE IN PAST 24 HOURS

PART D: OTHER MEDICINE

WAS ANY OTHER MEDICINE TAKEN THIS DAY? ☐ NO ☐ YES (IF YES, SPECIFY)

☐ VITAMINS ☐ IRON ☐ OTHER

PART E: DAY OF WEEK (CIRCLE CORRECT DAY FOR THIS DATE)

MONDAY TUESDAY WEDNESDAY THURSDAY FRIDAY SATURDAY SUNDAY

DATE: MONTH DAY YEAR

033

PLEASE BE SURE TO RETURN THIS DIARY CARD TO YOUR DOCTOR, AS WELL AS ALL TABLETS NOT TAKEN.

Address

Date

ATTACHMENT 2
CLINICAL RESEARCH PROTOCOL

Drug: Bendectin, each of its 3 ingredients alone and in combination, and placebo

Investigator: (if more than one, list all with senior first)

Objective: To evaluate the therapeutic efficacy of Bendectin, each of its 3 ingredients alone and in combination, and placebo in the management of nausea and/or vomiting during the first trimester of pregnancy. (This is part of a multi-clinic study.)

Where done and source of patients: Patients will be selected from practice of the principal investigator and his colleagues.

Plan of investigation: (Use extra pages if needed to describe in detail) This will be a double-blind study in 40 or more patients in which distribution of medication per patient will be randomized. The patient will be evaluated by the investigator at the initial visit and again following completion of 7 days treatment. The patient will complete a diary report form before treatment and on each of the 7 days of treatment, and will return these to the investigator immediately after the 7th day of treatment. (See attached protocol.)

Laboratory reports planned: (use extra pages if needed) None

Starting date:

Completion date

Procedure for reporting results:

Case report forms: (attach sample) See attached

Progress reports: (frequency and type) Physician's report and patient's diary forms to be sent to Merrell Monitor weekly as study progresses

Final report: (time and form) Sequential analysis of multi-clinic study by Merrell

Drug requirement estimate: (patient days, if dose unknown) 30 tablets per patient; 40 or more patients among whom 1/8 will receive Bendectin, 1/8 Bentyl, 1/8 Decapryn, 1/8 Bentyl/Decapryn, 1/8 pyridoxine, 1/8 Bentyl/pyridoxine, 1/8 Decapryn/pyridoxine, and 1/8 placebo.

(Merrell Project Director) (Senior Investigator)

No Carbon Required - Do Not Place On Other NCR Forms While Filling In

Do Not Write In This Space 614

NAUSEA AND VOMITING OF PREGNANCY

PHYSICIAN'S REPORT FORM

PROTOCOL NO.: INITIAL EVALUATION

PATIENT'S NAME: AGE:

PLEASE ATTACH TEAR-OFF PORTION OF APPROPRIATE MEDICATION LABEL HERE.

MARITAL STATUS: ☐ Married ☐ Single ☐ Other

RACE: ☐ Cauc. ☐ Negro ☐ Orient. ☐ Am. Ind. ☐ Other

GRAVIDA: (Including this pregnancy) ______

MEDICATION NUMBER

PARA: ABORTUS:

INITIAL EVALUATION: (Pretreatment) On Date Of Entering Study

TODAY'S DATE: Month Day Year

START OF LMP: Month Day Year

ESTIMATED DURATION OF GESTATION: (Weeks, as based on estimated date of conception)

TODAY'S WEIGHT: (lb.)

RECENT HISTORY OF PATIENT'S NAUSEA AND VOMITING OF PREGNANCY:

NAUSEA: ☐ None, vomiting only ☐ Yes - If yes, check both severity and duration.

SEVERITY OF NAUSEA: ☐ Mild ☐ Moderate ☐ Severe

DURATION OF NAUSEA: ☐ 1-2 Days ☐ 3-7 Days ☐ 8-14 Days ☐ More than 14 days

VOMITING: ☐ None, nausea on y ☐ Yes - If yes, check both severity and duration.

SEVERITY OF VOMITING: ☐ Mild ☐ Moderate ☐ Severe

DURATION OF VOMITING: ☐ 1-2 Days ☐ 3-7 Days ☐ 8-14 Days ☐ More th n 14 days

035

RETURN TO:
Medical Research Dept.
MERRELL-NATIONAL LABORATORIES
Division of Richardson-Merrell Inc.
Cincinnati, Ohio 45215

SIGNATURE ______ M. D.
NAME PRINTED
DATE

THIS COPY FOR MERRELL-NATIONAL LABORATORI

No Carbon Required - Do Not Place On Other NCR Forms While Filling In

615

NAUSEA AND VOMITING OF PREGNANCY

Do Not Write In This Space
1 DRUG CODE 2-3 CARD NUMBER 00

PHYSICIAN'S REPORT FORM

4-9 CASE NUMBER 10

PROTOCOL NO.: INITIAL EVALUATION

PATIENT'S NAME: AGE: 11-12

PLEASE ATTACH TEAR-OFF PORTION OF APPROPRIATE MEDICATION LABEL HERE.

13-MARITAL STATUS: 1 - ☐ Married 2 - ☐ Single 3 - ☐ Other 9

14-RACE: 1 - ☐ Cauc. 2 - ☐ Negro 3 - ☐ Orient.
4 - ☐ Am. Ind. 5 - ☐ Other 9

GRAVIDA: (including this pregnancy) 15-16 NS

MEDICATION NUMBER

PARA: 17-18 NS ABORTUS: 19-20 NS

INITIAL EVALUATION: (Pretreatment) On Date Of Entering Study

TODAY'S DATE: Month 21-22 Day 23-24 Year 25-26 NS

START OF LMP: Month 27-28 Day 29-30 Year 31-32 NS

ESTIMATED DURATION OF GESTATION: 33-34 NS (Weeks, as based on estimated date of conception)

TODAY'S WEIGHT: (lb.) 38-40 NS

RECENT HISTORY OF PATIENT'S NAUSEA AND VOMITING OF PREGNANCY:

41- 9 NAUSEA: 0 - ☐ None, vomiting only ☐ Yes - if yes, check both severity and duration.

SEVERITY OF NAUSEA: 1 - ☐ Mild 2 - ☐ Moderate 3 - ☐ Severe

42-9 DURATION OF NAUSEA: 1- ☐ 1-2 Days 2- ☐ 3-7 Days 3- ☐ 8-14 Days 4- ☐ More than 14 days

43- 9 VOMITING: 0 - ☐ None, nausea only ☐ Yes - if yes, check both severity and duration.

SEVERITY OF VOMITING: 1 - ☐ Mild 2 - ☐ Moderate 3 - ☐ Severe

44-9 DURATION OF VOMITING: 1- ☐ 1-2 Days 2- ☐ 3-7 Days 3- ☐ 8-14 Days 4- ☐ More than 14 days

036

50 51 52 53 54 55 56 57 58 59 60 61 62 63 64 65 66 67 68 69 70 71 72 73 74 75 AE 77-80

SIGNATURE
NAME PRINTED ____________ M. D.
DATE______

THIS COPY FOR MERRELL-NATIONAL LABORATORIES

NO CARBON REQUIRED - DO NOT PLACE ON OTHER NCR FORMS WHILE FILLING IN

No 0 [illegible]

616

DO NOT WRITE IN THIS SPACE

NAUSEA AND VOMITING OF PREGNANCY

PHYSICIAN'S REPORT FORM

PROTOCOL NO:

FINAL EVALUATION

PATIENT'S NAME: AGE: MEDICATION NUMBER:

FINAL EVALUATION: (TO BE COMPLETED AT END OF STUDY)

DATE OF PATIENT'S VISIT: MONTH: DAY: YEAR:

STUDY COMPLETED ACCORDING TO PROTOCOL CRITERIA?

☐ YES ☐ NO - IF NO, GIVE REASON: ☐ PATIENT DID NOT RETURN ☐ OTHER (SPECIFY)

TODAY'S WEIGHT: (LB.)

SEVERITY OF PATIENT'S NAUSEA: ☐ NONE ☐ MILD ☐ MODERATE ☐ SEVERE

SEVERITY OF PATIENT'S VOMITING: ☐ NONE ☐ MILD ☐ MODERATE ☐ SEVERE

HAS PATIENT TAKEN ANY OTHER MEDICATION DURING THIS TIME? ☐ NO ☐ YES (IF YES, SPECIFY)

	NAME	DOSE/DAY	DATES OF MEDICATION
☐ VITAMINS (SPECIFY)			
☐ IRON (SPECIFY)			
☐ HYPNOTIC (SPECIFY)			
☐ ANALGESIC (SPECIFY)			
☐ ANTIHISTAMINE (SPECIFY)			
☐ OTHER (SPECIFY)			

WERE ANY SIDE EFFECTS VOLUNTEERED BY PATIENT? ☐ NONE ☐ YES (LIST BELOW)

EFFECTIVENESS OF MEDICATION: ☐ EXCELLENT ☐ MODERATE ☐ SLIGHT ☐ NONE

OVERALL EVALUATION AND COMMENTS:

038

RETURN TO:
Medical Research Dept.
MERRELL-NATIONAL LABORATORIES
Division of Richardson-Merrell Inc.

SIGNATURE

NAME PRINTED M. D.

DATE

NO CARBON REQUIRED - DO NOT PLACE ON OTHER NCR FORMS WHILE FILLING IN

DO NOT WRITE IN THIS SPACE

NAUSEA AND VOMITING OF PREGNANCY

1 DRUG CODE 2-3 CARD NUMBER 01

PHYSICIAN'S REPORT FORM

4-9 CASE NUMBER 10

PROTOCOL NO: FINAL EVALUATION

PATIENT'S NAME: AGE: MEDICATION NUMBER:

11-12 13-15

FINAL EVALUATION: (TO BE COMPLETED AT END OF STUDY)

DATE OF PATIENT'S VISIT: MONTH: DAY: YEAR:

16-17 18-19 20-21 NS

STUDY COMPLETED ACCORDING TO PROTOCOL CRITERIA?

22 - 1 - ☐ YES 0 - ☐ NO - IF NO, GIVE REASON: 23- 1 - ☐ PATIENT DID NOT RETURN

2 - ☐ OTHER (SPECIFY)

TODAY'S WEIGHT: (LB.) (USE COLS., 40 - 71)

24-26 ØNS

27- SEVERITY OF PATIENT'S NAUSEA: 0 - ☐ NONE 1 - ☐ MILD 2 - ☐ MODERATE 3 - ☐ SEVERE 9

28- SEVERITY OF PATIENT'S VOMITING: 0 - ☐ NONE 1 - ☐ MILD 2 - ☐ MODERATE 3 - ☐ SEVERE 9

29- HAS PATIENT TAKEN ANY OTHER MEDICATION DURING THIS TIME? 0 - ☐ NO 1 - ☐ YES (IF YES, SPECIFY)

	NAME	DOSE/DAY	DATES OF MEDICATION
30 - 1- ☐ VITAMINS (SPECIFY)			
31 - 1- ☐ IRON (SPECIFY)			
32 - 1- ☐ HYPNOTIC (SPECIFY)			
33 - 1- ☐ ANALGESIC (SPECIFY)			
34 - 1- ☐ ANTIHISTAMINE (SPECIFY)			
35 - 1- ☐ OTHER (SPECIFY)			

I DEC SYSTEM

36- WERE ANY SIDE EFFECTS VOLUNTEERED BY PATIENT? 0 - ☐ NONE 1 - ☐ YES (LIST BELOW) 9

IDEC (STEM)

37- EFFECTIVENESS OF MEDICATION: 3 - ☐ EXCELLENT 2 - ☐ MODERATE 1 - ☐ SLIGHT 0 - ☐ NONE 9

OVERALL EVALUATION AND COMMENTS:

SE COLS. 40 - 71

10 41 42 43 44 45 46 47 48 49 50 51 52 53 54 55 56 57 58 59 60 61 62 63 64 65 66 67 68 69 70 71 72 73 74 75 AE

77-80

039

SIGNATURE

NAME PRINTED M. D.

DATE

NO CARBON REQUIRED - DO NOT PLACE ON OTHER NCR FORMS WHILE FILLING IN

DO NOT WRITE IN THIS SPACE

NAUSEA AND VOMITING OF PREGNANCY

PHYSICIAN'S REPORT FORM

PROTOCOL NO:

FINAL EVALUATION

PATIENT'S NAME:

AGE:

MEDICATION NUMBER:

FINAL EVALUATION: (TO BE COMPLETED AT END OF STUDY)

DATE OF PATIENT'S VISIT: MONTH: DAY: YEAR:

STUDY COMPLETED ACCORDING TO PROTOCOL CRITERIA?

☐ YES ☐ NO - IF NO, GIVE REASON: ☐ PATIENT DID NOT RETURN ☐ OTHER (SPECIFY)

TODAY'S WEIGHT: (LB.)

SEVERITY OF PATIENT'S NAUSEA: ☐ NONE ☐ MILD ☐ MODERATE ☐ SEVERE

SEVERITY OF PATIENT'S VOMITING: ☐ NONE ☐ MILD ☐ MODERATE ☐ SEVERE

HAS PATIENT TAKEN ANY OTHER MEDICATION DURING THIS TIME? ☐ NO ☐ YES (IF YES, SPECIFY)

	NAME	DOSE/DAY	DATES OF MEDICATION
☐ VITAMINS (SPECIFY)			
☐ IRON (SPECIFY)			
☐ HYPNOTIC (SPECIFY)			
☐ ANALGESIC (SPECIFY)			
☐ ANTIHISTAMINE (SPECIFY)			
☐ OTHER (SPECIFY)			

WERE ANY SIDE EFFECTS VOLUNTEERED BY PATIENT? ☐ NONE ☐ YES (LIST BELOW)

EFFECTIVENESS OF MEDICATION: ☐ EXCELLENT ☐ MODERATE ☐ SLIGHT ☐ NONE

OVERALL EVALUATION AND COMMENTS:

040

SIGNATURE

NAME PRINTED M. D.

DATE

BE SURE TO COMPLETE YOUR DIARY CARD EACH EVENING BEFORE TAKING YOUR MEDICATION.

DO NOT WRITE IN THIS SPACE 619

PATIENT'S DAILY DIARY CARD

PATIENT'S NAME:

DR.'S NAME:

STUDY DAY (CHECK ONE)

☐ IN DR.'S OFFICE BEFORE START OF MEDICATION (DO NOT FILL OUT PART C TODAY) (REMEMBER TO FILL OUT DAY-1 CARD TOMORROW EVENING)

☐ DAY 1 (DAY AFTER SEEING YOUR DOCTOR)

☐ DAY 2 ☐ DAY 3 ☐ DAY 4 ☐ DAY 5 ☐ DAY 6 ☐ DAY 7

PART A: NAUSEA

NAUSEA PRESENT ON THIS DAY? ☐ YES (IF YES, CHECK 1 BOX IN EACH OF THE 3 COLUMNS) ☐ NO (IF NO, GO DIRECTLY TO PART B)

1. DURATION
- ☐ LESS THAN 1 HOUR
- ☐ 1 TO 2 HOURS
- ☐ 3 TO 4 HOURS
- ☐ 5 TO 6 HOURS
- ☐ 7 TO 12 HOURS
- ☐ 13 TO 16 HOURS
- ☐ 17 TO 24 HOURS

2. EFFECT ON USUAL DAILY ACTIVITIES
- ☐ NONE
- ☐ SOMEWHAT REDUCED
- ☐ MUCH REDUCED
- ☐ UNABLE TO PERFORM ANY USUAL DAILY ACTIVITIES

3. EFFECT ON EATING
- ☐ NONE
- ☐ ATE SOMEWHAT LESS
- ☐ ATE MUCH LESS
- ☐ UNABLE TO EAT ANYTHING ALL DAY

(FOLD HERE)

PART B: VOMITING

VOMITING PRESENT ON THIS DAY? ☐ YES (IF YES, CHECK 1 BOX IN EACH OF THE 2 COLUMNS) ☐ NO (IF NO, GO DIRECTLY TO PART C)

1. TYPE OF VOMITING
- ☐ GAGGING ONLY
- ☐ DRY HEAVING
- ☐ SOME VOMITING
- ☐ HEAVY VOMITING

2. FREQUENCY
- ☐ 1 TIME THIS DAY
- ☐ 2 TIMES THIS DAY
- ☐ 3-5 TIMES THIS DAY
- ☐ 6-12 TIMES THIS DAY
- ☐ MORE THAN 12 TIMES THIS DAY

PART C: MEDICINE FOR NAUSEA AND VOMITING DURING PAST 24 HOURS

(CHECK EACH TIME THE MEDICINE WAS TAKEN)

☐ 2 TABLETS LAST NIGHT BEFORE GOING TO BED ☐ 1 TABLET THIS MORNING ☐ 1 TABLET THIS AFTERNOON ☐ NONE IN PAST 24 HOURS

PART D: OTHER MEDICINE

WAS ANY OTHER MEDICINE TAKEN THIS DAY? ☐ NO ☐ YES (IF YES, SPECIFY)

☐ VITAMINS ☐ IRON ☐ OTHER

PART E: DAY OF WEEK (CIRCLE CORRECT DAY FOR THIS DATE)

MONDAY TUESDAY WEDNESDAY THURSDAY FRIDAY SATURDAY SUNDAY

DATE: MONTH DAY YEAR 041

PLEASE BE SURE TO RETURN THIS DIARY CARD TO YOUR DOCTOR AS WELL AS ALL TABLETS NOT TAKEN.

BE SURE TO COMPLETE YOUR DIARY CARD EACH EVENING BEFORE TAKING YOUR MEDICATION/
NO DEJE VD. DE COMPLETAR SU CARTA DIARIA CADA TARDE ANTES DE TOMAR SU MEDICACION

620

PATIENT'S DAILY DIARY CARD/CARTA DIARIA DEL PACIENTE

Do not write in this space/
No escribir en este espacio

PATIENT'S NAME/NOMBRE DEL PACIENTE

DOCTOR'S NAME/NOMBRE DEL MEDICO

STUDY DAY (Check one)/DIA DEL ESTUDIO (Indicar uno)

☐ IN DR.'S OFFICE BEFORE START OF MEDICATION
(Do not fill out PART C today)
(Remember to fill out day-1 card tomorrow evening)/
EN EL CONSULTORIO DEL MEDICO ANTES DE INICIARSE LA MEDICACION
(No completar PARTE C hoy)
(No olvidar de completar mañana por la tarde su carta diaria para día-1)

☐ DAY 1 (Day after seeing your doctor)/
DIA 1 (Día después de consultar Su médico)

☐ DAY/DIA 2 ☐ DAY/DIA 5
☐ DAY/DIA 3 ☐ DAY/DIA 6
☐ DAY/DIA 4 ☐ DAY/DIA 7

PART/PARTE A: NAUSEA/NAUSEA

NAUSEA PRESENT ON THIS DAY?/
¿NAUSEA PRESENTE EN ESTE DIA?

☐ YES/SI (If yes, check 1 box in each of the 3 columns/Si la respuesta es sí, poner una cruz en 1 caja en cada una de las 3 columnas)

☐ NO/NO (If no, go directly to PART B/Si no, proceder directamente a PARTE B

1. DURATION/DURACION
- ☐ LESS THAN 1 HOUR/MENOS DE 1 HORA
- ☐ 1 TO 2 HOURS/1 A 2 HORAS
- ☐ 3 TO 4 HOURS/3 A 4 HORAS
- ☐ 5 TO 6 HOURS/5 A 6 HORAS
- ☐ 7 TO 12 HOURS/7 A 12 HORAS
- ☐ 13 TO 16 HOURS/13 A 16 HORAS
- ☐ 17 TO 24 HOURS/17 A 24 HORAS

2. EFFECT ON USUAL DAILY ACTIVITIES/ EFECTO SOBRE ACTIVIDADES DIARIAS ACOSTUMBRADAS
- ☐ NONE/NINGUN EFECTO
- ☐ SOMEWHAT REDUCED/ALGO REDUCIDAS
- ☐ MUCH REDUCED/MUY REDUCIDAS
- ☐ UNABLE TO PERFORM ANY USUAL DAILY ACTIVITIES/TODAS ACTIVIDADES DIARIAS ACOSTUMBRADAS IMPOSIBLES

3. EFFECT ON EATING/ EFECTO SOBRE EL COMER
- ☐ NONE/NINGUN EFECTO
- ☐ ATE SOMEWHAT LESS/ ALGO MENOS COMIDO
- ☐ ATE MUCH LESS/MUCHO MENOS COMIDO
- ☐ UNABLE TO EAT ANYTHING ALL DAY/ TODO COMER IMPOSIBLE DURANTE TODO EL DIA

(FOLD HERE/DOBLAR AQUI)

PART/PARTE B: VOMITING/VOMITO

VOMITING PRESENT ON THIS DAY?/
¿VOMITO PRESENTE EN ESTE DIA?

☐ YES/SI (If yes, check 1 box in each of the 2 columns/Si la respuesta es sí, poner una cruz en 1 caja en cada una de las 2 columnas)

☐ NO/NO (If no, go directly to PART C/Si no, proceder directamente a PARTE C)

1. TYPE OF VOMITING/CARACTER DEL VOMITO
- ☐ GAGGING ONLY/ASCO SOLAMENTE
- ☐ DRY HEAVING/VOMITO SECO
- ☐ SOME VOMITING/ALGUN VOMITO
- ☐ HEAVY VOMITING/VOMITO ABUNDANTE

2. FREQUENCY/FREQUENCIA
- ☐ 1 TIME THIS DAY/1 VEZ EN ESTE DIA
- ☐ 2 TIMES THIS DAY/2 VECES EN ESTE DIA
- ☐ 3-5 TIMES THIS DAY/3-5 VECES EN ESTE DIA
- ☐ 6-12 TIMES THIS DAY/6-12 VECES EN ESTE DIA
- ☐ MORE THAN 12 TIMES THIS DAY/MAS DE 12 VECES EN ESTE DIA

PART/PARTE C: MEDICINE FOR NAUSEA AND VOMITING DURING PAST 24 HOURS
MEDICAMENTO PARA NAUSEA Y VOMITO DURANTE LAS ULTIMAS 24 HORAS

(Check each time the medicine was taken/Indicar cada vez que se tomó el medicamento)

☐ 2 TABLETS LAST NIGHT BEFORE GOING TO BED/ 2 PASTILLAS ANOCHE ANTE DE ACOSTARSE

☐ 1 TABLET THIS MORNING/ 1 PASTILLE HOY POR LA MAÑANA

☐ 1 TABLET THIS AFTERNOON/ 1 PASTILLA HOY POR LA TARDE

☐ NONE IN PAST 24 HOURS/NINGUN MEDICAMENTO DURANTE LAS ULTIMAS 24 HORAS

PART/PARTE D: OTHER MEDICINE/OTRA MEDICACION

WAS ANY OTHER MEDICINE TAKEN THIS DAY?/
¿SE TOMO OTRA MEDICACION EN ESTE DIA?

☐ NO/NO ☐ YES/SI (If yes, specify/Si la respuesta es sí, particularizar)

☐ VITAMINS/ VITAMINAS ☐ IRON/ HIERRO ☐ OTHER/ OTRAS

042

PART/PARTE E: DAY OF THE WEEK (Circle correct day for this date)/
DIA DE LA SEMANA (Circundar día apropiado a esta fecha)

MONDAY LUNES	TUESDAY MARTES	WEDNESDAY MIERCOLES	THURSDAY JUEVES	FRIDAY VIERNES	SATURDAY SABADO	SUNDAY DOMINGO

DATE/FECHA: MONTH/MES ______ DAY/DIA ______ YEAR/ANO

PLEASE BE SURE TO RETURN THIS DIARY CARD TO YOUR DOCTOR, AS WELL AS ALL TABLETS NOT TAKEN./

Bendectin

NDA-10-598

(DESI 10598)

Dicyclomine/Doxylamine (Bentyl/Decapryn)

(IND 7620)

Summary of Reported Adverse Reactions--"8-way" Study

Comparative adverse reaction information for the 2308 tabulated patients of drug-treated (dicyclomine--Bentyl; doxylamine--Decapryn; dicyclomine/doxylamine--Bentyl/Decapryn; pyridoxine; dicyclomine/pyridoxine--Bentyl/pyridoxine; doxylamine/pyridoxine--Decapryn/pyridoxine; or Bendectin) and placebo-treated groups is presented in the accompanying summary Tables A through I. For this study, no adverse reactions were solicited; all adverse reactions here considered were volunteered by the patients. The data in these summary tables were derived from IBM print-outs #927 and #901 (Volume 4). Copies of the individual Drug Experience Reports (Form FD-1639) are in Volume 5.

Of the 2308 patient population data base, 2158 patients had data completed with regard to adverse reactions. A total of 319 adverse reactions were reported among 258 (12.0%) of these 2158 patients. Tables A, B, and C, respectively, list the 49 different adverse reactions reported with percents calculated as follows:

Table A. Percents based on number of patients with adverse reaction data completed (per medication group).

Table B. Percents based on number of patients reporting 1 or more adverse reactions (per medication group).

3/14/75

Table C. Percents based on number of adverse reactions reported (per medication group).

The percent of patients volunteering 1 or more adverse reactions ranged from 8.7% for doxylamine/pyridoxine (Decapryn/pyridoxine) to 15.2% for doxylamine (Decapryn) among the medication groups as follows, with the placebo incidence of 11.2% and dicyclomine/pyridoxine (Bentyl/pyridoxine) incidence of 12.4% approximating the 12.0% mean of the total population (calculated from Table A):

Medication Group	% of Patients
Doxylamine/pyridoxine (Decapryn/pyridoxine)	8.7
Pyridoxine	9.6
Dicyclomine (Bentyl)	10.7
Placebo	11.2
Dicyclomine/pyridoxine (Bentyl/pyridoxine)	12.4
Dicyclomine/Doxylamine (Bentyl/Decapryn)	13.4
Bendectin	14.3
Doxylamine (Decapryn)	15.2
Mean of Total Population	12.0

Of the 49 reported different adverse reactions (Tables A, B, and C), the 3 adverse reactions of highest incidence (>1% of patients) were drowsiness, fatigue, and headache (Tables A and D). The 74 reports of drowsiness occurred more frequently among the 4 patient groups receiving doxylamine (Decapryn) than in the placebo-treated group or the other 3 drug-treated groups (Table D).

Forty-eight (18.6%) of the 258 patients volunteered more than 1 adverse reaction (Table E). Only 9 (3.5%) of the 258 patients reported more than 2 symptoms, and these 9 were distributed among 6 of the drug-treated (Bendectin excluded) and the placebo groups.

The percentage incidence of the different adverse reactions reported among the 7 groups of drug-treated patients was never more than 3% above that of the placebo-treated group (Tables F and G). Table F is a summary listing of the 6 reported adverse reactions for which a percentage incidence was >1% above that of the placebo-treated patients, and Table G is a summary listing of the 3 reported adverse reactions for which a percentage incidence was >1% below that of the placebo-treated patients. Patient groups taking medication which contained doxylamine (Decapryn) had an incidence of drowsiness from 1.56% to 2.69% above placebo, whereas patients taking dicyclomine (Bentyl), pyridoxine, or a combination of dicyclomine/pyridoxine (Bentyl/pyridoxine) had an incidence of drowsiness of 1.49% to 1.86% below placebo. The incidence of drowsiness among placebo patients was 2.97%. Headache had a 1.34% to 2.37% greater incidence compared to placebo among patients taking dicyclomine (Bentyl), dicyclomine/doxylamine (Bentyl/Decapryn), or dicyclomine/pyridoxine (Bentyl/pyridoxine), whereas all other medication groups showed an incidence of headache of <1% difference from the placebo incidence of 1.49%.

Table H shows, by investigator, the number and percentage of tabulated patients reporting 1 or more adverse reactions and arranged in decreasing magnitude of incidence, with a range from 40% to 0%.

3/14/75

Overall, with regard to volunteered adverse reactions, drowsiness was a symptom which could be related to an ingredient on the basis of comparative incidence among the drug-treated and placebo groups. Patient groups receiving 1 of the 4 medications containing doxylamine (Decapryn) had an incidence of drowsiness ranging from 4.53% to 5.66%, compared to the incidence in placebo patients of 2.97%. The lowest incidence of the total of volunteered adverse reactions was in the group receiving doxylamine/pyridoxine (Decapryn/pyridoxine), and this incidence was 2.5% below that of the placebo group, and 3.3% below the mean of the total population.

Untabulated Patients

Of the 51 untabulated patients, there was record of 12 patients (1 of 12 uncertain) having taken medication. Of these 12, only 1 volunteered an adverse reaction. This patient reported dizziness. The medication taken by this patient was not identifiable (label lost and medication number not entered on case report form).

Comment

The slightly greater incidence over placebo of patients volunteering 1 or more adverse reactions among those receiving doxylamine (Decapryn) with or without dicyclomine (Bentyl) was observed in this "8-way" study as well as in the previously completed "4-way" study (see Table I). The incidence of adverse reactions when solicited in the "4-way" study was considerably higher. The slightly greater incidence over placebo of volunteered adverse reactions among patients receiving Bendectin was roughly comparable for this "8-way" study and the previously completed Bendectin vs. placebo "2-way" study. The

incidence of placebo-treated patients volunteering 1 or more adverse reactions in the 3 sequential multi-center studies was approximately the same (10.6% to 11.2%).

3/14/75

TABLE A

BENDECTIN ANTINAUSEANT 8-WAY STUDY -- VOLUNTEERED ADVERSE REACTIONS
319 ADVERSE REACTIONS REPORTED AMONG 258 PATIENTS FOR A POPULATION OF 2308 PATIENTS

Percent based on number of patients with adverse reaction data completed.

ADVERSE REACTIONS REPORTED	Bentyl #/%	Decapryn #/%	Bentyl/ Decapryn #/%	Pyridoxine #/%	Bentyl/ Pyridoxine #/%	Decapryn/ Pyridoxine #/%	Bendectin #/%	Placebo #/%	Total
Abdominal Bloating or Flatulence			1/0.35		1/0.38	1/0.38	1/0.38	1/0.37	5/0.23
Abdominal Cramps			1/0.35	1/0.37					2/0.09
Abdominal Pa[illegible]						1/0.38			1/0.05
Allergic Reaction, Ill-Defined	2/0.74	2/0.74		4/1.48	1/0.38	1/0.38	1/0.38	5/1.86	16/0.74
[illegible]ia	1/0.37			1/0.37	1/0.38				3/0.13
Appetite Increase		1/0.37		1/0.37	1/0.38		1/0.38		[illegible]0.18
[illegible]	1/0.37			1/0.37	1/0.38		1/0.38		4/0.18
Breast Change (pain, soreness)	1/0.37						1/0.38		2/0.09
Breathing Difficulties, NOS	1/0.37								1/0.05
Chest Pain or Rib Cage Pain		1/0.37		1/0.37					2/0.09
Concentration - Difficulty, Disturbance							1/0.38		1/0.05
Constipation								1/0.37	1/0.05
Depression, Mild to Moderate or NOS	1/0.37								1/0.05
Diarrhea	1/0.37	1/0.37				1/0.38		3/1.12	6/0.28
Dizziness		4/1.49	4/1.41	1/0.37	5/1.88	3/1.13	1/0.38	2/0.74	20/0.93
[illegible] Drowsiness; Sleepy	4/1.49	14/5.20	15/5.30	3/1.11	3/1.13	15/5.66	12/4.53	8/2.97	74/3.43
Dry [illegible]	1/0.37		2/0.71	1/0.37	2/0.75		1/0.38		7/0.32
[illegible] (includes 1 Dysphagia of Rx+)					1/0.38	1/0.38†			2/0.09
[illegible]		1/0.37			1/0.38				2/0.09
Faint Feeling, [illegible]	2/0.74	1/0.37	3/1.06						6/0.28
Fatigue (Tiredness) or Lethargy	4/1.48	[illegible]/2.23	7/2.47	1/0.37	5/1.88	2/0.75	5/1.89	3/1.12	33/1.53
Gastric Irritation; Heartburn; Indigestion	3/1.11	2/0.74		2/0.74		2/0.75			9/0.42
[illegible] (includes 1 migraine†)	9/3.32	6/2.23	8/2.83	5/1.85	10/3.76+	2/0.75	4/1.51	4/1.49	48/2.22
[illegible]				1/0.37		1/0.38	1/0.38	1/0.37	4/0.18
Irritability		1/0.37					1/0.38		2/0.09
[illegible]				1/0.37					1/0.05
[illegible]								1/0.37	1/0.05
[illegible]	2/0.74	2/0.74			1/0.38		3/1.13		[illegible]0.37
[illegible]	1/0.37								1/0.05
Nervousness or Tension	3/1.11	1/0.37	1/0.35	2/0.74			1/0.38	2/0.74	10/0.46
[illegible]								1/0.37	2/0.09
[illegible]		1/0.37	1/0.35	1/0.37	1/0.38		3/1.13	1/0.37	7/0.32
[illegible] Effect					1/0.38				1/0.05
[illegible] Sensitivity [illegible]							1/0.38		1/0.05
Spotting or Bleeding during Pregnancy			1/0.35		1/0.38				2/0.09
Stools, Change in Color, Consistency			1/0.35						1/0.05
[illegible]		1/0.[illegible]	1/0.35	1/0.37					3/0.1[illegible]
Taste Alteration[illegible]							1/0.38	1/0.37	2/0.09
Temperature Rise						1/0.38			1/0.05
[illegible]						1/0.38			1/0.05
Throat, [illegible]		2/0.74							2/0.09
[illegible]					2/0.75				2/0.09
[illegible]	1/0.37		2/0.71						3/0.13
[illegible]				1/0.37					1/0.05
Visual Complaints - Blurring of Vision		1/0.37							1/0.05
Visual Complaints - Lights, Floaters				1/0.37					1/0.05
Weakness, Generalized or NOS			4/1.41	1/0.37			2/0.75		7/0.32
[illegible]					2/0.75				2/0.09
[illegible] of Symptoms	1/0.37				1/0.38				2/[illegible]
No. Adverse Reactions Reported	39	48	52	31	41	32	42	34	319
No. Patients Reporting 1 or More	29	41	38	26	33	23	3[illegible]	30	256
No. Patients with Data Completed	271	269	283	270	266	265	265	269	2158
No. Patients with Data Not Stated	2	4	3	2	2	2	2	1	15
No. Patients [illegible] Not Return	13	14	15	18	18	17	21	[illegible]	132
[illegible] Patients Enrolled	286	287	301	290	286	284	[illegible]	[illegible]86	2[illegible]

NOS = Not Otherwise Stated

**NEC = Not Elsewhere Classified

3/14/75

TABLE 8

BENDECTIN ANTINAUSEANT 8-WAY STUDY -- VOLUNTEERED ADVERSE REACTIONS
319 ADVERSE REACTIONS REPORTED AMONG 258 PATIENTS FOR A POPULATION OF 2308 PATIENTS

Percents based on number of patients reporting 1 or more adverse reactions.

ADVERSE REACTIONS REPORTED	Bentyl #/%	Decapryn #/%	Bentyl/ Decapryn #/%	Pyridoxine #/%	Bentyl/ Pyridoxine #/%	Decapryn/ Pyridoxine #/%	Bendectin #/%	Placebo #/%	Total
Abdominal Bloating or Flatulence			1/02.63		1/03.03	1/04.35	1/02.63	1/03.33	5/01.94
Abdominal Cramps			1/02.63	1/03.85					2/00.78
Abdominal Pain						1/04.35			1/00.39
Adverse Reaction, Ill-Defined	2/06.90	2/04.88		4/15.38	1/03.03	1/04.35	1/0[illegible].63	5/16.67	16/06.20
[illegible]	1/03.45			1/03.85	1/03.03				3/01.16
Appetite Increase		1/02.44		1/03.85	1/03.03		1/02.63		4/01.55
[illegible]	1/03.45			1/03.85	1/03.03		1/02.63		4/01.55
Breast Changes (pain, soreness)	1/03.45						1/02.63		2/00.78
Breathing Difficulties, NOS*	1/03.45								1/00.39
Chest Pain or Rib Cage Pain		1/02.44		1/03.85					2/00.78
Concentration - Difficulty, Disturbance							1/02.63		1/00.39
Constipation								1/03.33	1/00.39
Depression, Mild to Moderate or NOS*	1/03.45								1/00.39
Diarrhea	1/03.45	1/02.44				1/04.35		3/10.00	6/02.32
Dizziness		4/09.75	4/10.53	1/03.85	5/15.15	3/13.04	1/02.63	2/06.67	20/07.75
[illegible], Drowsiness, Sleep	4/13.79	14/34.15	15/39.47	3/11.54	3/09.09	15/65.22	12/31.58	8/26.66	74/28.69
Dry Mouth	1/03.45		2/05.26	1/03.85	2/06.06		1/02.63		7/02.71
[illegible] (includes 1 [illegible] of Eye)					1/03.03	1/04.35			2/00.78
Epistaxis		1/02.44			1/03.03				2/00.78
Faint [illegible]; Presyncope	2/06.90	1/02.44	3/07.89						6/02.32
Fatigue [illegible] or Lethargy	4/13.79	6/14.63	7/18.42	1/03.85	5/15.15	2/08.70	5/13.16	3/10.00	33/12.79
Gastric Irritation; Heartburn, Indigestion	3/10.34	2/04.88		2/07.69		2/08.70			9/03.49
Headache (includes 1 migraine)	9/31.03	6/14.63	8/21.05	5/19.23	10/30.30	2/08.70	4/10.53	4/13.33	[illegible]
Insomnia				1/03.85		1/04.35	1/02.63	1/03.33	4/01.55
Irritability		1/02.44					1/02.63		2/00.78
Itching, Skin				1/03.85					1/00.39
[illegible]								1/03.33	1/00.39
Lightheadedness	2/06.90	2/04.88			1/03.03		3/07.89		8/03.10
[illegible]	1/03.45								1/00.39
Nervousness or Tension	3/10.34	1/02.44	1/02.63	2/07.69			1/02.63	2/06.67	[illegible]
[illegible]								1/03.33	[illegible]
[illegible]		1/02.44	1/02.63	1/03.85	1/03.03		3/07.89	1/03.33	[illegible]
[illegible] Effect					1/03.03				1/00.39
Sensations, [illegible]							1/02.63		1/00.39
Spotting or [illegible] during Pregnancy			1/02.63		1/03.03				2/00.78
Stool, Change in Color, Consistency			1/02.63						1/00.39
[illegible]		1/02.44	1/02.63	1/03.85					[illegible]
Taste Alterations							1/02.63	1/03.33	[illegible]
[illegible]						1/04.35			[illegible]
Thirst						1/04.35			[illegible]
[illegible], NOS*		2/04.88							[illegible]
Tinnitus					2/06.06				[illegible]
[illegible]	1/03.45		2/05.26						[illegible]
[illegible]				1/03.85					[illegible]
Visual Complaints - Blurring of Vision		1/02.44							[illegible]
Visual Complaints - Lights, Floaters				1/03.85					[illegible]
[illegible] NEC**			4/10.53	1/03.85			2/05.[illegible]		[illegible]
[illegible]					2/06.06				[illegible]
[illegible]	1/0[illegible].45				1/03.03				[illegible]
No. Adverse Reactions Reported	39	48	52	31	41	32	42	34	319
No. Patients Reporting 1 or More	[illegible]	41	38	26	33	[illegible]	[illegible]	30	258
No. Patients with Data for Listed	[illegible]	[illegible]	283	270	266	265	265	269	2156
[illegible] Data Not Stated	2	4	3	2	2	2	2	[illegible]	15
[illegible] Not Return	13	24	15	16	13	17	21	16	[illegible]
[illegible] Enrolled	[illegible]	[illegible]	301	[illegible]	266	284	[illegible]	[illegible]	[illegible]

*NOS = Not Otherwise Stated

**NEC = Not Elsewhere Classified

3/14/75

TABLE C

BENDECTIN ANTINAUSEANT 8-WAY STUDY -- VOLUNTEERED ADVERSE REACTIONS
319 ADVERSE REACTIONS REPORTED AMONG 258 PATIENTS FOR A POPULATION OF 2308 PATIENTS

Percents based on number of adverse reactions reported.

ADVERSE REACTIONS REPORTED	Bentyl #/%	Decapryn #/%	Bentyl/ Decapryn #/%	Pyridoxine #/%	Bentyl/ Pyridoxine #/%	Decapryn/ Pyridoxine #/%	Bendectin #/%	Placebo #/%	Total
Abdominal Bloating or Flatulence			1/01.92		1/02.44	1/03.12	1/07.38	1/02.94	5/01.57
Abdominal Cramps			1/01.92	1/03.22					[illegible]
Abdominal Pain						1/03.12			1/00.31
[illegible] Reaction, Ill-defined	2/05.13	2/04.17		4/12.90	1/02.44	1/03.12	1/02.38	5/14.70	[illegible]
Anorexia	1/02.56			1/03.22	1/02.44				3/00.94
Appetite Increase		1/02.08		1/03.22	1/02.44		1/02.38		4/01.25
Backache	1/02.56			1/03.22	1/02.44		1/02.38		4/01.25
Breast (tenderness, pain, soreness)	1/02.56						1/02.38		2/00.63
Breathing Difficulties, NOS*	1/02.56								1/00.31
Chest Pain or Rib Cage Pain		1/02.08		1/03.22					2/00.63
Concentration - Difficulty, Disturbance							1/02.38		1/00.31
Constipation								1/02.94	1/00.31
Depression, Mild to Moderate or NOS*	1/02.56								1/00.31
Diarrhea	1/02.56	1/02.08				1/03.12		3/08.82	6/01.88
Dizziness		4/08.33	4/07.69	1/03.22	5/12.20	3/09.38	1/02.38	2/05.88	20/06.27
Drowsiness; Sleepy	4/10.26	14/29.17	15/28.85	3/09.68	3/07.32	15/46.88	12/28.57	8/23.53	74/23.20
Dry Mouth	1/02.56		2/03.85	1/03.22	2/04.88		1/02.38		7/02.19
Dysphagia (includes 1 Dysphagia of Rx*)					1/02.44	1/03.12			2/00.63
[illegible]is		1/02.08			1/02.44				2/00.63
Faint Feeling; Syncope	2/05.13	1/02.08	3/05.77						6/01.88
Fatigue (tiredness) or Lethargy	4/10.26	6/12.50	7/13.46	1/03.22	5/12.20	2/06.25	5/11.90	3/08.82	33/10.34
Gastric Irritation, Heartburn; Indigestion	3/07.69	2/04.17		2/06.45		2/06.25			9/02.82
Headache (includes 1 migraine*)	9/23.08	6/12.50	8/15.38	5/16.13	10/24.39	2/06.25	4/09.52	4/11.76	[illegible]
Insomnia				1/03.22		1/03.12	1/02.38	1/02.94	4/01.25
Irritability		1/02.08					1/02.38		2/00.63
Itching Skin				1/03.22					1/00.31
Lassitude								1/02.94	1/00.31
Light-headedness	2/05.13	2/04.17			1/02.44		3/07.14		8/02.51
[illegible]	1/02.56								1/00.31
Nervousness or Tension	3/07.69	1/02.08	1/01.92	2/06.45			1/02.38	2/05.88	[illegible]
[illegible]						1/03.12		1/02.94	[illegible]
[illegible]		1/02.08	1/01.92	1/03.22	1/02.44		3/07.14	1/02.94	7/02.19
No Drug Effect					1/02.44				1/00.31
[illegible]							1/02.38		1/00.31
[illegible] Bleeding during Pregnancy			1/01.92		1/02.44				2/00.63
Stools - [illegible] color, Consistency			1/01.92						1/00.31
[illegible]		1/02.08	1/01.92	1/03.22					[illegible]
Taste [illegible]							1/02.38	1/02.94	[illegible]
[illegible]						1/03.12			[illegible]
[illegible]						1/03.12			1/00.31
Throat, Sore, NOS*		2/04.17							[illegible]
[illegible]					2/04.88				2/00.63
[illegible]	1/02.56		2/03.85						3/00.94
[illegible]				1/03.22					1/00.31
Visual Complaints - Blurring of Vision		1/02.08							1/00.31
Visual Complaints - [illegible]				1/03.22					[illegible]
[illegible] Generalized [illegible]			4/07.69	1/03.22			2/04.76		7/02.19
[illegible]					2/04.88				2/00.63
[illegible]	1/02.56				1/02.44				2/00.63
No. Adverse Reactions Reported	39	48	52	31	41	32	42	34	319
No. Patients Reporting 1 or More	29	41	38	26	33	23	33	30	[illegible]
No. Patients with Data Completed	271	269	283	270	266	265	265	259	[illegible]
No. Patients with Data Not Stated	2	4	3	2	2	2	2	1	[illegible]
No. Patients [illegible] Did Not Return	13	14	15	18	18	17	21	16	[illegible]
No. Patients Enrolled	286	287	301	290	286	284	288	[illegible]	[illegible]

*NOS = Not Otherwise Stated

**NEC = Not Elsewhere Classified

3/14/75

TABLE D

Bendectin Antinauseant 8-Way Study

Volunteered Adverse Reactions Showing an Overall Incidence

of 1% or More from a Population of 2158* Patients

Adverse Reaction Reported	Bentyl #/%	Decapryn #/%	Bentyl/ Decapryn #/%	Pyridoxine #/%	Bentyl/ Pyridoxine #/%	Decapryn/ Pyridoxine #/%	Bendectin #/%	Placebo #/%	Total
Drowsy; Drowsiness; Sleepy	4/05.40	14/15.92	15/20.27	3/04.05	3/04.05	15/20.27	12/16.22	8/10.81	74
Fatigue (Tiredness) or Lethargy	4/12.12	6/18.18	7/21.21	1/03.03	5/15.15	2/06.06	5/15.15	3/09.09	33
Headache (includes 1 migraine†)	9/18.75	6/12.50	8/16.67	5/10.42	10/20.83†	2/04.17	4/08.33	4/08.33	48
Total	17	26	30	9	18	19	21	15	155

*From total population of 2308 patients; excludes no returns and data not stated
†Percents based on total patients/adverse reaction

TABLE E

Bendectin Antinauseant 8-Way Study

Number of Adverse Reactions Reported per Patient

Volunteered - Population of 2158* Patients

No. Adverse Reactions	Bentyl	Decapryn	Bentyl/ Decapryn	Pyridoxine	Bentyl/ Pyridoxine	Decapryn/ Pyridoxine	Bendectin	Placebo	Total
1	22	35	28	22	26	17	34	26	210
2	6	5	9	3	4	5	4	3	39
3		1	2	1	1			1	6
4					1	1			2
5	1								1
Total Patients	29	41	39	26	32	23	38	30	258

*No. patients with data completed from a total population of 2308 patients

3/14/75

TABLE F

Bendectin Antinauseant 8-Way Study

Adverse Reactions with a Frequency of > 1% Above Placebo

Volunteered - Population of 2158* Patients

Adverse Reaction	Bentyl #/%	Decapryn #/%	Bentyl/ Decapryn #/%	Pyridoxine #/%	Bentyl/ Pyridoxine #/%	Decapryn/ Pyridoxine #/%	Bendectin #/%	Placebo #/%
Drowsy; Drowsiness; Sleepy		14/5.20	15/5.30			15/5.66	12/4.53	8/2.97
Faint Feeling; Presyncope			3/1.06					0
Gastric Irritation; Heartburn; Indigestion	3/1.11							0
Headache (includes 1 migraine†)	9/3.32		8/2.83		10/3.76†			4/1.49
Lightheadedness							3/1.13	0
Weakness, Generalized or NOS**			4/1.41					0

*No. patients with data completed from Table A
**NOS = Not Otherwise Stated

TABLE G

Bendectin Antinauseant 8-Way Study

Adverse Reactions with a Frequency of > 1% Below Placebo

Volunteered - Population of 2158* Patients

Adverse Reaction	Bentyl #/%	Decapryn #/%	Bentyl/ Decapryn #/%	Pyridoxine #/%	Bentyl/ Pyridoxine #/%	Decapryn/ Pyridoxine #/%	Bendectin #/%	Placebo #/%
Adverse Reaction Ill-Defined	2/0.74	2/0.7[illegible]	0		1/0.38	1/0.38	1/0.38	5/1.8[illegible]
Diarrhea			0	0	0		0	3/1.12
Drowsy; Drowsiness; Sleepy	4/1.48			3/1.11	3/1.13			8/2.97

*No. patients with data completed from Table A

3/14/75

TABLE II

Bendectin Antinauseant 8-Way Study

Incidence of Patients with 1 or More Adverse Reactions per Investigator

Arranged in Decreasing Magnitude

and Expressed as % ot Each Investigator's Population with Data Completed

Investigator No.	Investigator Name	No. Pts. with Data Completed	No. Pts. Reporting 1 or More Adverse Reactions	%
120	McCarty	5	2	40.00
115	Daily	39	13	33.33
133	Rush	63	20	31.75
122	Taylor	79	24	30.38
132	Rowley	92	22	23.91
138	Wendt	39	8	20.51
121	Weed	77	13	17.10
118	Malinak	61	10	16.39
116	Crisp	27	4	14.81
130	Kritzer	89	13	14.61
129	Iffy	59	7	13.56
135	Salter	77	10	12.99
117	Greer	71	9	12.68
114	Greenblatt	73	9	12.33
124	Cefalo	57	6	12.28
137	Kuhn	132	16	12.12
127	DerYuen	26	3	11.54
101	Cameron	152	17	11.18
123	Wallach	9	1	11.11
136	DeRuiter	114	10	8.77
119	McQuarrie	153	13	8.50
131	Robison	156	12	7.69
111	Phillips	81	6	7.41
100	Berry	57	3	5.26
125	Rivo	36	1	2.78
128	Celniker	126	2	1.57
112	Bolding	129	2	1.55
104	Ficklen	41	0	0.00
113	Balin	38	0	0.00
All Investigators		2158	258	11.96

3/14/75

Table I

Bendectin Antinauseant "8-Way" Study

Comparison Among "2-Way," "4-Way," and "8-Way" Studies of Incidence of Patients Volunteering Adverse Reactions

Medication Group	% of patients volunteering 1 or more adverse reactions		
	Bendectin vs. placebo "2-way"[a,b] study	Bentyl/ Decapryn "4-way"[a,c] study	Bendectin "8-way"[b] study
Doxylamine/pyridoxine (Decapryn/pyridoxine)			8.7
Pyridoxine			9.6
Dicyclomine (Bentyl)		12.7(56.6)[d]	10.7
Placebo	10.9	10.6(58.3)[d]	11.2
Dicyclomine/pyridoxine (Bentyl/pyridoxine)			12.4
Dicyclomine/doxylamine (Bentyl/Decapryn)		18.3(57.3)[d]	13.4
Bendectin	14.8		14.3
Doxylamine (Decapryn)		20.4(55.4)[d]	15.2

[a] from Volume 31 of submission of 12/15/72

[b] study involved only volunteered adverse reactions

[c] study involved volunteered and solicited adverse reactions

[d] % of patients with 1 or more adverse reactions solicited (submission of 12/15/72)

Listing of Investigators

STUDY PROTOCOL 008- -004

Bendectin, Each of Its Three Ingredients Alone and in Combination, and [illegible]

Investigator No.	Investigator Name	Investigator Location	Status	Tabulated Case Repor[illegible]
113	Balin, H.	Philadelphia, PA	(In progress)	39
100	Berry, F.X.	Greensboro, NC	(Terminated)	59
112	Bolding, O.T.	Birmingham, AL	"	143
101	Cameron, W.J.	Kansas City, KS	"	174
1[illegible]4	Cefalo, R.C.	Bethesda, MD	(In progress)	60
128	Colniker, B.	Phoenix, AZ	(Terminated)	131
116	Crisp, W.	Phoenix, AZ	(In progress)	29
115	Daily, L.	Houston, TX	(Terminated)	39
136	DeRuiter, J.W.	Winter Haven, FL	"	117
127	DorYuen, D.	Seattle, WA	"	31
104	Ficklen, C.	Wilmington, NC	"	43
114	Greenblatt, R.	Augusta, GA	"	73
117	Greer, B.E.	Denver, CO	"	74
129	Iffy, L.	Philadelphia, PA	"	66
130	Kritzer, L.	Dayton, OH	"	91
137	Kuhn, M.J.	Milwaukee, WI	"	144
118	Malinak, L.R.	Houston, TX	"	61
120	McCarty, W.D.	Albuquerque, NM	"	5
119	McQuarrie, H.G.	Salt Lake City, UT	"	161
111	Phillips, W.	Fort Smith, AR	"	[illegible]6
125	Rivo, E.	Boston, MA	"	38
131	Robison, R.D.	Idaho Falls, ID	"	16[illegible]
132	Rowley, D.A.	Mesa, AZ	"	[illegible]
133	Rush, R.E.	Pocatello, ID	"	6[illegible]
135	Salter, J.I.	Fairfield, OH	"	8[illegible]
122	Taylor, D L.	Lakeland, FL	"	9[illegible]
123	Wallach, E.	Philadelphia, PA	"	[illegible]
121	Weed, J.C.	New Orleans, LA	"	8[illegible]
138	Wendt, W [illegible]	Milwaukee, WI	"	[illegible]
			TOTAL	23[illegible]

Study Protocol 008- -004

Drug Distribution Schedule

Bendectin, Each of its Three Ingredients Alone and in Combination, and Placebo ("8-Way")

Investigator			Drug		
No.	Name	Location	Index No.	Medication No.	
113	Balin, H.	Philadelphia, PA	261<br>270	1-48<br>49-96	
100	Berry, F.X.	Greensboro, NC	265	1-72	
112	Bolding, O.T.	Birmingham, AL	268	1-144	
101	Cameron, W.J.	Kansas City, KS	264<br>278	1-96<br>101-196	
124	Cefalo, R.C.	Bethesda, MD	275<br>276	1-96<br>149-196	
128	Calniker, B.	Phoenix, AZ	324	1-192	5/10/73
116	Crisp, W.	Phoenix, AZ	267	1-96	1/16/73
115	Daily, L.	Houston, TX	262	1-48	12/27/72
136	DeRuiter, J.W.	Winter Haven, FL	360	1-120	7/5/73
127	DorXuen, D.	Seattle, WA	318	1-48	[illegible]
104	Ficklan, C.	Wilmington, NC	270	1-48	
114	Greenblatt, R.	Augusta, GA	263	1-96	
117	Greer, B.E.	Denver, CO	266	1-96	
129	Iffy, L.	Philadelphia, PA	276	1-48<br>97-120	
130	Kritzer, L.	Dayton, OH	320	1-96	
137	Kuhn, M.J.	Milwaukee, WI	326	1-192	
118	Malinak, L.R.	Houston, TX	274	1-96	
120	McCarty, W.D.	Albuquerque, NM	272	1-96	
119	McQuarrie, H.G.	Salt Lake City, UT	273<br>277	1-144<br>149-196	
108	*Montz, C.E.	Bismarck, ND	315	1-45	
111	Phillips, W.	Fort Smith, AR	269	1-96	
125	Rivo, E.	Boston, MA	317	1-48	
131	Robison, R.D.	Idaho Falls, ID	318<br>322	97-192<br>1-96	
132	Rowley, D.A.	Mesa, AZ	323	1-96	
133	Rush, R.E.	Pocatello, ID	321	1-72	
126	*Salerno, L.J.	New York, NY	316	1-48	
135	Salter, J.I.	Fairfield, OH	319	1-96	
134	*St. Clair, P.J.	Cleveland, OH	280	1-96	
122	Taylor, D.L.	Lakeland, FL	279	1-96	
123	Wallach, E.	Philadelphia, PA	277	1-48	
121	Weed, J.C.	New Orleans, LA	271	1-96	
138	Wendt, W.P.	Milwaukee, WI	325	1-48	

* Terminated (never started--all drug returned)
