# Supplementary material for: 8-Way Randomized Controlled Trial of Doxylamine, Pyridoxine and Dicyclomine for Nausea and Vomiting during Pregnancy: Restoration of Unpublished Information
Source: PLoS One. 2017 Jan 4;12(1):e0167609. doi: 10.1371/journal.pone.0167609 (PMC5215753; doi:10.1371/journal.pone.0167609)
Supplement: S3 Appendix — A tool based on the CONSORT 2010 checklist, for documenting the transformation from regulatory documents to journal publication. (DOCX) [file pone.0167609.s004.docx]

RIAT Audit Record (RIATAR)

*A tool for documenting the transformation from regulatory documents to journal publication, based on the CONSORT 2010 checklist of information to include when reporting a randomised trial**

| Section/Topic | Item No | Checklist item | Reported on page No of RIAT manuscript | Source section(s) of the Clinical Study Report (CSR): page No. and paragraph | PDF page No (for PDF files) | Notes |
| --- | --- | --- | --- | --- | --- | --- |
| Title and abstract | | | | Page 606, “procedure” section | Protocol, page 3 |  |
|  | 1a | Identification as a randomised trial in the title | Page1-3 |  |  |  |
|  | 1b | Structured summary of trial design, methods, results, and conclusions (for specific guidance see CONSORT for abstracts) | Page 1-3 | Page 582, “summary of ‘8-way- multi-center study” section  Page 583, “summary of protocol”  Page 584, “summary of results”  Page 588, “conclusion” | Report, pages 22, 23, 24, 28 respectively |  |
| Introduction | | | |  |  |  |
| Background and objectives | 2a | Scientific background and explanation of rationale | Page 5-6 | Page 567, “rationale for treatment” | Report, page 6 |  |
|  | 2b | Specific objectives or hypotheses | Page 6 | Page 605, “objective” | Protocol, page 2 |  |
| Methods | | | |  |  |  |
| Trial design | 3a | Description of trial design (such as parallel, factorial) including allocation ratio | Page 6-10 | Unable to obtain information | Unable to obtain information | Unable to obtain information |
|  | 3b | Important changes to methods after trial commencement (such as eligibility criteria), with reasons | Page 14 | Page 590, “re-evaluation omitting data from Dr Balin” | Report, page 30 | The reason for this omission is documented in a letter from Alexander M. Schmidt, Commisioner of Food and Drugs which was not obtained. |
| Participants | 4a | Eligibility criteria for participants | Page 8 | Page 605, “Patients” | Protocol, Page 2 |  |
|  | 4b | Settings and locations where the data were collected | Page 8 | Page 605, “Patients” | Protocol, page 2 | The study report states “14 clinics”, further details of the location were not disclose. |
| Interventions | 5 | The interventions for each group with sufficient details to allow replication, including how and when they were actually administered | Page 8 | Page 606, “Medications” | Protocol, page 3 |  |
| Outcomes | 6a | Completely defined pre-specified primary and secondary outcome measures, including how and when they were assessed | Page 8-9 | Page 608, “Evaluation of results and statistical analysys”, paragraph 1  Page 606, “Procedure”, paragraphs 1-5 | Protocol, page 3-4 |  |
|  | 6b | Any changes to trial outcomes after the trial commenced, with reasons | Unable to obtain information | Unable to obtain information | Unable to obtain information | Unable to obtain information |
| Sample size | 7a | How sample size was determined | Unable to obtain information | Unable to obtain information | Unable to obtain information | Unable to obtain information |
|  | 7b | When applicable, explanation of any interim analyses and stopping guidelines | Unable to obtain information | Unable to obtain information | Unable to obtain information | Unable to obtain information |
| Randomisation: |  |  |  |  |  |  |
| Sequence generation | 8a | Method used to generate the random allocation sequence | Page 9 | Page 606, “Procedure”, | Protocol, page 3-5 | Not explicitly disclosed, but one can infer that a centralised service at Merrell-National Laboratories was used. |
|  | 8b | Type of randomisation; details of any restriction (such as blocking and block size) | Unable to obtain information | Unable to obtain information | Unable to obtain information | Unable to obtain information |
| Allocation concealment mechanism | 9 | Mechanism used to implement the random allocation sequence (such as sequentially numbered containers), describing any steps taken to conceal the sequence until interventions were assigned | Page 9 | Page 607, “Procedure”, paragraph 2. | Protocol, page 4 |  |
| Implementation | 10 | Who generated the random allocation sequence, who enrolled participants, and who assigned participants to interventions | Unable to obtain information | Unable to obtain information | Unable to obtain information | Unable to obtain information, likely implemented by investigators and merrell- National Project Monitor. |
| Blinding | 11a | If done, who was blinded after assignment to interventions (for example, participants, care providers, those assessing outcomes) and how | Page 9 | Page 606, “Procedure”, paragraph 1,2. | Protocol, page 3, 4 |  |
|  | 11b | If relevant, description of the similarity of interventions | Page 8 | Page 606, “Medications” | Protocol, page 3 |  |
| Statistical methods | 12a | Statistical methods used to compare groups for primary and secondary outcomes | Page 10 | Unable to obtain information | Unable to obtain information | The study report states the data was subjected to statistical analysis by the biostatical department of Merrell-National Laboratories. The biostatical report (volume 3) was not obtainable. |
|  | 12b | Methods for additional analyses, such as subgroup analyses and adjusted analyses | Unable to obtain information | Unable to obtain information | Unable to obtain information | The study report states the data was subjected to statistical analysis by the biostatical department of Merrell-National Laboratories. The biostatical report (volume 3) was not obtainable. |
| Results | | | |  |  |  |
| Participant flow (a diagram is strongly recommended) | 13a | For each group, the numbers of participants who were randomly assigned, received intended treatment, and were analysed for the primary outcome | Page 10-11 | Page 584, “Summary of Results”, paragraph 1  Page 595 (all minus Balin’s patients), 596 (with Balin’s patients), | Report, pages 24, 35 (all minus Balin’s patients), 36 (with Balin’s patients), | No diagrams were included in the study report |
|  | 13b | For each group, losses and exclusions after randomisation, together with reasons | Page 10-11 | Page 596-603 | Report, pages 35-43 | The reasons for losses and exclusions were not explicitly identified in the study report |
| Recruitment | 14a | Dates defining the periods of recruitment and follow-up | Page 11 | Page 590, “Current status” | Report, pages 30 | The original data was received on July 22 1974. A re-evaluation omitting data from Dr. Balin was done on April 1, 1975. The period of recruitment and follow-up were not specified in the study report. |
|  | 14b | Why the trial ended or was stopped | Unable to obtain information | Unable to obtain information | Unable to obtain information | Unable to obtain information |
| Baseline data | 15 | A table showing baseline demographic and clinical characteristics for each group | Unable to obtain information | Unable to obtain information | Unable to obtain information | Unable to obtain information |
| Numbers analysed | 16 | For each group, number of participants (denominator) included in each analysis and whether the analysis was by original assigned groups | Page 10-11 | Unable to obtain information | Unable to obtain information | Unable to obtain information |
| Outcomes and estimation | 17a | For each primary and secondary outcome, results for each group, and the estimated effect size and its precision (such as 95% confidence interval) | Page 11 | Page 592-593 | Report, pages 32-33 | P values are “one sided probability”, likely referring to a one-tailed test. |
|  | 17b | For binary outcomes, presentation of both absolute and relative effect sizes is recommended | Unable to obtain information | Unable to obtain information | Unable to obtain information | Unable to obtain information |
| Ancillary analyses | 18 | Results of any other analyses performed, including subgroup analyses and adjusted analyses, distinguishing pre-specified from exploratory | Unable to obtain information | Unable to obtain information | Unable to obtain information | Unable to obtain information |
| Harms | 19 | All important harms or unintended effects in each group (for specific guidance see CONSORT for harms) | Page 13-14 | Pages 621-634, “Summary of Reported Adverse Reactions” | Protocol, pages 18-31 |  |
| Discussion | | | |  |  |  |
| Limitations | 20 | Trial limitations, addressing sources of potential bias, imprecision, and, if relevant, multiplicity of analyses | Page 14-15 | Unable to obtain information | Unable to obtain information | Unable to obtain information |
| Generalisability | 21 | Generalisability (external validity, applicability) of the trial findings | Page 14-15 | Unable to obtain information | Unable to obtain information | Unable to obtain information |
| Interpretation | 22 | Interpretation consistent with results, balancing benefits and harms, and considering other relevant evidence | Page 15-16 | Pages 588-590, “Conclusions” | Report, pages 28-30 |  |
| Other information | | |  |  |  |  |
| Registration | 23 | Registration number and name of trial registry | Page 7 | Page 621 | Protocol, page 18 | The study report discloses its NDA, DESI and IND number |
| Protocol | 24 | Where the full trial protocol can be accessed, if available | Supplementary file | Pages 604-620 | Protocol, pages 1-17 |  |
| Funding | 25 | Sources of funding and other support (such as supply of drugs), role of funders | Page 17 |  |  |  |

* The aim of this audit tool is provide a permanent record of the parts of text, tables and figures of the source Clinical Study Report (CSR) selected for inclusion into the RIAT manuscript submitted for publication. This tool is based upon checklist items described in the CONSORT 2010 statement, which is a widely adopted standard for reporting randomised trials. RIAT authors should consult the CONSORT 2010 Explanation and Elaboration for important clarifications on all the items. Similar audit records can be created for other types of trials by adapting other CONSORT extensions, e.g. for cluster randomised trials, non-inferiority and equivalence trials, non-pharmacological treatments, herbal interventions, and pragmatic trials. See [www.consort-statement.org](http://www.consort-statement.org) for more details.
